# Supplementary material for: A tool for modeling gene regulatory networks (GRN_modeler) and its applications to synthetic biology
Source: Mol Syst Biol. 2025 Sep 29;21(11):1618–37. doi: 10.1038/s44320-025-00148-8 (PMC12583811; doi:10.1038/s44320-025-00148-8)
Supplement: Supplementary file 1 — Appendix [file 44320_2025_148_MOESM1_ESM.pdf]

Appendix for

A Tool for Modeling Gene Regulatory Networks (GRN_modeler) and its Applications to Synthetic Biology

Gábor Holló[1*], Jung Hun Park[1], Emanuele Boni[1], Yolanda Schaerli[1*]

[1]Department of Fundamental Microbiology, University of Lausanne, Biophore Building, 1015, Lausanne, Switzerland

Contents

1 Manual **3**
1.1 Command line functionality . . . 3
1.2 Representing Interactions . . . 5
1.3 Spatial simulations . . . 7
1.3.1 Numerical methods . . . 7
1.3.2 Further simulation details related to Figure 10 . . . 7
1.3.3 Intracellular diffusion . . . 7
1.4 Manual for the Graphical User Interface . . . 8
1.5 Available solvers . . . 11

2 Simulations **13**
2.1 Simulation time . . . 13
2.2 Elowitz-model . . . 14
2.3 Tomazou-model . . . 15
2.4 CRISPR model . . . 16
2.5 The coherent and incoherent feed-forward loop . . . 17
2.6 Implementing systems with self-regulating nodes . . . 18
2.7 Finding steady states in the toggle switch . . . 18
2.8 Novel oscillator circuits . . . 19
2.8.1 The "actolator" family . . . 19
2.8.2 Modified repressilator circuits: the "acrelator" family . . . 21
2.9 Light biosensor . . . 24

3 Experiments **26**

*Correspondence E-mail: gabor.hollo@unil.ch, yolanda.schaerli@unil.ch

List of Appendix Figures

Appendix Figure S1 5
Appendix Figure S2 7
Appendix Figure S3 13
Appendix Figure S4 17
Appendix Figure S5 18
Appendix Figure S6 18
Appendix Figure S7 19
Appendix Figure S8 20
Appendix Figure S9 20
Appendix Figure S10 22
Appendix Figure S11 23
Appendix Figure S12 24
Appendix Figure S13 25
Appendix Figure S14 26
Appendix Figure S15 27
Appendix Figure S16 28
Appendix Figure S17 29

List of Appendix Tables

Appendix Table S1 13
Appendix Table S2 14
Appendix Table S3 14
Appendix Table S4 15
Appendix Table S5 16
Appendix Table S6 24
Appendix Table S7 29

1 Manual

1.1 Command line functionality

- `Cell('ModelName')`: Instantiates a new object for the gene regulatory network specified by `'ModelName'`. This name should match one of the models implemented in the **models** folder. The currently available models are "Elowitz," "Tomazou," and "CRISPR," as described in the Results section. For example, use `Ecoli = ... Cell('Elowitz');` to create a new instance of the "Elowitz" model with the user-defined name "Ecoli".

- `add_node('NodeName', 'NodeType')`: This method adds a new node to the system with the specified `'NodeName'` and `'NodeType'`. If only one node type is defined in the model, specifying the type is optional, and a simplified naming convention is used for parameters that do not include the type. This simplification also applies to proteases and regulators. For example, use `Ecoli = Ecoli.add_node('N1', 'type1');` to add a node named 'N1' with type 'type1' to the `Ecoli` model.

- `add_protease('NodeName', 'ProteaseName')`: Adds a protease, specified by `'ProteaseName'`, to the node identified as `'NodeName'`. For example, use `Ecoli = Ecoli.add_protease('N1', 'PROT1', 'type1');` to add the protease `'PROT1'` to the node `'N1'` in the `Ecoli` model. The purpose of this function is to introduce an additional species that can interact with existing species across different nodes. We demonstrate this feature using a protease as an example, but it could also be applied to RNA or other species.

- `add_regulator('RegulationType', 'obj_name', 'obj_input', 'reg_name1')`: Adds a regulator to a specified object. In Appendix Figure S1 we show the general concept of the regulation. The regulation type is defined by `'RegulationType'`. The name of the regulated object is `'obj_name'`, and its selected input is `'obj_input'`, which is regulated by `'reg_name1'`. For example, to add a `'Repression_out'` type regulation to the `'N1'` node through its input named `'HILL'`, regulated by the `'R1'` species (`N1HILL|-R1`), use: `Ecoli = ... Ecoli.add_regulator('Repression_out', 'N1', 'HILL', 'R1');`. To add regulators to existing regulations, we can extend the input list using the following syntax: `add_regulator('RegulationType', 'obj_name', ... 'obj_input', 'reg_name1', 'reg_input1', 'reg_name2')`. Here, `'reg_input1'` specifies the input of the first regulator (`'reg_name1'`), while `'reg_name2'` is the name of the new regulator. For example, to add another regulator (`'R2'`) to the existing regulation (`N1HILL|-R1`) with a type `'Activation_out'` for the `'HILL'` input (`N1HILL|-R1HILL<-R2`), use: `Ecoli = Ecoli.add_regulator('Activation_out', 'N1', 'HILL', 'R1', ... 'HILL', 'R2');`. If only one input function is implemented in the model, this can be simplified to: `Ecoli = ... Ecoli.add_regulator('Activation_out', 'N1', 'R1', 'R2');`. If there is only one input, the naming process will be simplified both in the graph and in the variable names, resulting in a representation like `N1|-R1<-R2`. Regulators can have an unlimited number of hierarchical levels by further extending the input list in the same way. To simplify this process, one can utilize the code generation feature of the GUI.

- `set('Parameter_name', 'Property', Value, 'object_name')`: Sets the property (`'Property'`) of a specified parameter (`'Parameter_name'`) to a given value (`Value`) within a specific object (`'object_name'`). If the parameter belongs to a regulator, you can extend the input list with additional regulator names as needed. For example, `Ecoli.set('P_N2', 'InitialAmount', 100, 'N2');` sets the initial amount of the species `'P_N2'` to `100` for the `'N2'` node.

- `get('Parameter_name', 'Property', 'object_name')`: This method retrieves the property (`'Property'`) of a specified parameter (`'Parameter_name'`) for a given object (`'object_name'`). The syntax is similar to the `set` method, but the `Value` is the output rather than the property being set. For example, `Value = ... Ecoli.get('P_N2', 'InitialAmount', 'N2');` retrieves the initial amount of the parameter `'P_N2'` for the object `'N2'`.

- `make_graph()`: Generates a graph for the GRN, where the nodes, proteases, and regulators are the "dots" of the graph, and the interactions are represented by arrows in the directed graph. For example, use `Ecoli.make_graph();` to create the graph for the `Ecoli` model.

- `get_model()`: Creates a SimBiology model object. This model can be opened with the SimBiology application for analysis or to run simulations. For example, use `Mobj = Ecoli.get_model();` to create a model object from the `Ecoli` instance.

- `[t, c, names] = run_simulation(model, solver)`: This function runs a simulation on the specified `model` using the `solver`. If the `solver` is a COPASI solver rather than a built-in MATLAB solver, this command can be used similarly to MATLAB's `sbiosimulate` function. Here, `t` represents the output time points, `c` is a matrix of concentrations, and `names` contains the species names. The simulation length, tolerance values, and tracked species can be configured using SimBiology's built-in functionalities, such as the `getconfigset` function. To run a stochastic simulation, the reactions should be rewritten to create a COPASI-compatible SBML file:

```
configset = getconfigset(Mobj); %Retrieve the simulation settings from the model
Mobj = convert2irrev(Mobj); %Convert reversible reactions into two irreversible reactions
Mobj = correct_modifiers(Mobj); %Rewrite the reactions to make them COPASI compatible
Ecoli.set_configset(Mobj, configset); %Apply the original configuration settings
[t, c, names] = run_simulation(Mobj, 'adaptivesa'); %Run the simulation with the selected ...
adaptivesa solver
```

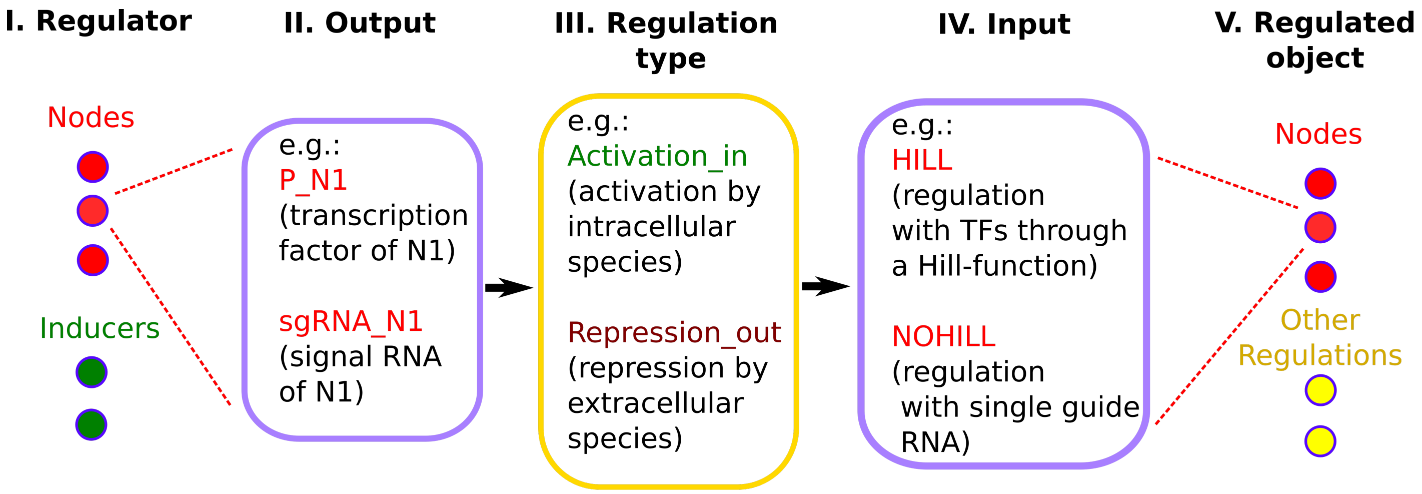

Appendix Figure S1: The basic concept and structure for setting up the regulatory system. Regulators (such as nodes or inducers like L-arabinose) can have multiple outputs (e.g., transcription factors or sgRNA) to control different targets. After selecting the appropriate regulation type, the regulator can be connected to a regulated object (another node or regulation) via its input, forming the regulatory network.

1.2 Representing Interactions

In this section, we outline how regulatory interactions are interpreted and implemented in our application.

Simple Regulation Consider a reaction where mRNA is transcribed and regulated by transcription factors: $\emptyset \longleftrightarrow \mathrm{mRNA}$. If the reaction rate follows the form (see Appendix Table S3):

$$\mathrm{r} = \mathrm{k}_0 + \mathrm{k}_1 \cdot \mathrm{HILL}([\mathrm{P}]), \tag{1}$$

where k_0 and k_1 are parameters, and [P] represents the concentration of a transcription factor, we can describe this regulation using a `rule` in SimBiology. For instance, a Hill function can be defined as:

$$\mathrm{HILL}([\mathrm{P}]) = \frac{1}{1 + ([\mathrm{P}]/\mathrm{K})^n}, \tag{2}$$

where K is the half saturation constant and n is the Hill exponent. This function can be any MATLAB-compatible function, not just Hill functions. If a different regulatory interaction is needed, users can modify the predefined "Rules" located in the `models` folder of the application.

Regulating a Regulator In many cases, small inducible molecules modulate the strength of an interaction. To incorporate this, we structured the regulation rules so that the activity or concentration of a regulator can itself be influenced by another Hill function. For example:

$$\mathrm{HILL}([\mathrm{P}]) = \frac{1}{1 + ([\mathrm{P}] \cdot \mathrm{HILL}_{2}/\mathrm{K}_{\mathrm{P}})^n}, \tag{3}$$

where HILL_2 can be defined as another regulatory function, such as repression by AHL:

$$\mathrm{HILL}_2([\mathrm{AHL}]) = \frac{1}{1 + ([\mathrm{AHL}] \cdot \mathrm{HILL}_{3/}\mathrm{K}_{\mathrm{AHL}})^n} \tag{4}$$

This approach allows for cascading regulatory interactions of arbitrary depth, though in biological systems, regulatory chains typically remain limited to a few layers.

Multiple Regulators For multiple regulators, the simplest case arises when the regulatory interactions can be expressed as a product of individual regulations (e.g., $\mathrm{HILL} = \mathrm{HILL}_1 \cdot \mathrm{HILL}_2$). In this scenario, no special multivariable functions are required, as the interactions can be automatically handled by the application. We illustrate this with several basic examples in Appendix Figure S2. Specifically, we demonstrate how the application can automatically generate regulatory functions for double activation (Equation 5), a combination of activation and inhibition (Equation 6), double inhibition (Equation 7), and a case with two repressions and one activation (Equation 8):

$$\mathrm{HILL}([\mathrm{P}_{\mathrm{N2}}], [\mathrm{P}_{\mathrm{N3}}]) = \frac{([\mathrm{P}_{\mathrm{N2}}]/\mathrm{K})^n}{1 + ([\mathrm{P}_{\mathrm{N2}}]/\mathrm{K})^n} \cdot \frac{([\mathrm{P}_{\mathrm{N3}}]/\mathrm{K})^n}{1 + ([\mathrm{P}_{\mathrm{N3}}]/\mathrm{K})^n}, \tag{5}$$

$$\mathrm{HILL}([\mathrm{P}_{\mathrm{N2}}], [\mathrm{P}_{\mathrm{N3}}]) = \frac{([\mathrm{P}_{\mathrm{N2}}]/\mathrm{K})^n}{1 + ([\mathrm{P}_{\mathrm{N2}}]/\mathrm{K})^n} \cdot \frac{1}{1 + ([\mathrm{P}_{\mathrm{N3}}]/\mathrm{K})^n}, \tag{6}$$

$$\mathrm{HILL}([\mathrm{P}_{\mathrm{N2}}], [\mathrm{P}_{\mathrm{N3}}]) = \frac{1}{1 + ([\mathrm{P}_{\mathrm{N2}}]/\mathrm{K})^n} \cdot \frac{1}{1 + ([\mathrm{P}_{\mathrm{N3}}]/\mathrm{K})^n}, \tag{7}$$

$$\mathrm{HILL}([\mathrm{P}_{\mathrm{N2}}], [\mathrm{P}_{\mathrm{N3}}], [\mathrm{P}_{\mathrm{N4}}]) = \frac{1}{1 + ([\mathrm{P}_{\mathrm{N2}}]/\mathrm{K})^n} \cdot \frac{1}{1 + ([\mathrm{P}_{\mathrm{N3}}]/\mathrm{K})^n} \cdot \frac{([\mathrm{P}_{\mathrm{N4}}]/\mathrm{K})^n}{1 + ([\mathrm{P}_{\mathrm{N4}}]/\mathrm{K})^n}, \tag{8}$$

where K and n represent the half-saturation constant and the Hill exponent, respectively. These parameters can be specific to each interaction; however, for simplicity, we use a common value here. P_{N2}, P_{N3}, P_{N4} denote the transcription factors.

However, in some cases, more complex interactions are required that cannot be expressed as a simple product of regulatory terms. For example, in our experiments, we worked with a system inducible by both light and arabinose, where multiple regulators influenced the same target species. We found that a more sophisticated equation was necessary to accurately capture the competitive nature of this dual activation process (see Equation 1 in the manuscript and Appendix Figure S13 for a visualization of these interactions). To implement such equations, modifications must be made to the “Rules” governing interactions in the `models` folder of the application. This flexible framework allows for the incorporation of any multivariable regulatory function using SimBiology “Rules,” enabling precise modeling of complex biological interactions.

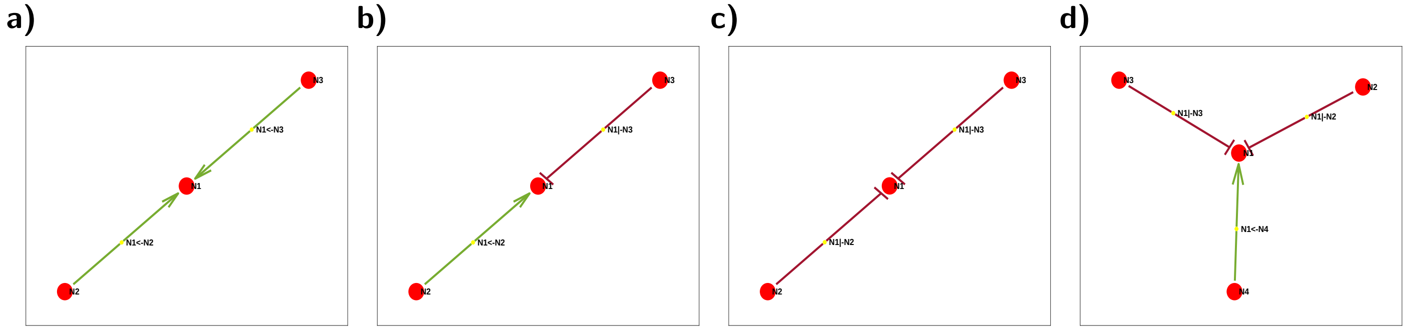

Appendix Figure S2: Examples of multiple regulators acting on a single node when the regulation can be expressed as a product of individual regulatory terms. (a) Two activators regulating a single node (Equation 5). (b) One activator and one repressor regulating a single node (Equation 6). (c) Two repressors regulating a single node (Equation 7). (d) A combination of two repressors and one activator regulating a single node (Equation 8).

1.3 Spatial simulations

1.3.1 Numerical methods

In the spatial simulations, we employed the finite difference method. The diffusion equation was solved using the Alternating Direction Implicit (ADI) method, where explicit and implicit approaches alternate along the two spatial coordinates. This method is unconditionally stable, allowing for larger time steps while still providing an approximate solution. For the implicit step, we utilized LU decomposition, significantly accelerating the solution of the linear system of equations involved in diffusion. For the diffusion of intracellular species in a growing colony (Equation 5), we applied the forward time-centered space (FTCS) scheme. The reaction kinetics terms were integrated using the Euler method.

1.3.2 Further simulation details related to Figure 10

The activator species had a diffusion coefficient of $D_{\text{act}} = 1 \text{ min}^{-1}$, while the inhibitor diffused faster with $D_{\text{inh}} = 10 \text{ min}^{-1}$. All other species were assumed immobile. To ensure nonlinear behavior, a Hill exponent of $n = 6$ was used. The system was initialized after 1000 minutes of reaction kinetics simulation, with an additional random perturbation applied to each species' initial concentration: $c^0_{i,\text{new}} = (1 + r)c^0_i$ where r is a uniformly distributed random number between -0.005 and 0.005, and c^0_i is the original initial concentration of species i. The half saturation constant for the self repression in N_2 was set to 55 molecule. We used a two-dimensional Gaussian function (amplitude 1, standard deviation 3) as the initial condition to represent a small initial colony. For both simulations, we used the Elowitz model, a system size of $L = 100$, spatial resolution $h = 0.5$, time step $dt = 0.1$ min, a total simulation time of 1000 minutes and periodic boundary conditions.

1.3.3 Intracellular diffusion

The description of diffusion of intracellular species that move together with the cells poses an interesting challenge how to describe the diffusion of intracellular species that move together with the cells. Here, we present a simplified derivation based on (Park et al, 2024; Cao et al, 2016).

The diffusion of the cells (denoted by c) can be described using the diffusion equation:

$$\frac{\partial c}{\partial t} = -\nabla \vec{j}_c = \nabla D_c \nabla c = D_c \nabla^2 c, \tag{9}$$

where D_c is the diffusion coefficient of the cells. The first equality corresponds to the continuity equation, which ensures mass conservation. The flux of the cells is given by $\vec{j}_c = -D_c \nabla c$, meaning that the driving force of cell movement is the gradient in cell concentration (∇c). In the final step, we assume that D_c is constant in space. Similarly, the total amount of an intracellular species is proportional to ca, where a is the concentration of the species inside a single cell. Since ca is a conserved quantity, its continuity equation is $\frac{\partial (ca)}{\partial t} = -\nabla \vec{j}_{ca}$. We assume that the flux of ca is proportional to the flux of cells and the intracellular concentration, so $\vec{j}_{ca} = a \vec{j}_c = -D_c a \nabla c$. This yields:

$$\boxed{\frac{\partial (ca)}{\partial t} = D_c \nabla a \nabla c.} \tag{10}$$

Now, using Equation 9, we obtain:

$$\frac{\partial (ca)}{\partial t} = D_c \nabla a \cdot \nabla c + D_c a \nabla^2 c = D_c \nabla a \cdot \nabla c + a \frac{\partial c}{\partial t}. \tag{11}$$

Finally, applying the product rule, $\frac{\partial (ca)}{\partial t} = c\frac{\partial a}{\partial t} + a\frac{\partial c}{\partial t}$, and rearranging, we get:

$$\boxed{\frac{\partial a}{\partial t} = D_c \nabla a \cdot \frac{\nabla c}{c}.} \tag{12}$$

This equation describes the effective diffusion of intracellular species that move with the cells. Notably, the diffusion is governed by the cell diffusion coefficient D_c, as the intracellular species do not diffuse independently but are transported passively along with the cells. It is important to emphasize that a itself is not conserved – only the product ca is conserved throughout the process.

1.4 Manual for the Graphical User Interface

This short instruction manual provides a quick reference to the key commands and steps needed for designing, analyzing, and exporting gene regulatory networks with the app GRN_modeler.

1. Opening the App and Creating a Gene Regulatory Network

   - Launch GRN_modeler – Open the GRN_modeler app. Note: requires Matlab 2022b or later.
   - Select Node Model – Choose one of the available node models (Elowitz, Tomazou, Javier, CRISPR. . .). *Example: select the Tomazou model if you want to account for transcription factor interactions and protein degradation.*
   - Add Nodes – In the Node Properties box (top right) click to add nodes. You can assign custom names or use default (N_1, N_2, . . .). To delete a node, select it and hit delete. *Note: in the context of Gene Regulatory Networks, each node represents a regulatory unit.*
   - Set Node Type and Properties – Use the Type and Set buttons to customize parameters (see Adjust Parameter Settings). *Note: you can select different types only if the selected node model contains more than one node type. For example, the Tomazou model only contains the transcription factor type.*

- Define Interactions – In the Regulator Properties box (center right) choose a regulator and target node; select input function and interaction type. To remove interactions, click on them and hit delete. *Example: to represent a transcription factor-based repression from N_1 to N_2, select P_N_1 (the protein product of node N_1) as regulator, N_2 as regulated element, Hill as input function, and* Repression_in *as interaction type. Note: you can add regulators different from nodes, for example chemical inducers. In this case, we recommend you specify the name of your inducer (e.g., Arabinose) as regulator, and select* Activation_out *or* Repression_out *as interaction type, to specify the regulator is external to the system. Note: besides nodes, you can select an interaction as regulated element (e.g., you can repress a repression).*
- Add Proteases – In the Protease Properties box (bottom right) click Add to include protease and account for protein degradation. You can assign a name or use the default (PROT#) and specify which nodes each protease regulates.
- Save Model – Click Input/Output then Save and assign a name to your network. This preserves your work for later editing.
- Adding External Models — Input/Output → Load External Model: Users can import models in the standard SBML format or as a SimBiology project file (".sbproj" extension), even if they are not based on our node-based structure. These models can represent any reaction kinetics system that interacts with specific species in our model. This functionality enables seamless integration with existing models, allowing for enhanced flexibility and reuse of previously developed systems. The entire reaction system can be viewed together using the "Open in SimBiology" option, which we introduce below.

2. Changing Graphical Settings and Parameter Settings

- Load Existing Model – Click Input/Output, then Load, and select your saved model. It automatically loads with the node model used during design.
- Customize Graph Settings – Under Settings → Graph Settings, adjust node size, colours (you can use colour names or RGB), font size, and choose a layout type (default is force). You can switch to an alternative representation by turning the 'Use the original graph' option ON. You can always reset to the default setting by clicking on Load default.
- Adjust Parameter Settings – For any component (node, regulator, or protease), click on it then press Set to modify parameters (initial amounts, kinetic values, etc.). *Note: only certain fields are editable (e.g., mRNA, unfolded/folded protein amounts; k_mat for maturation...). Example: you can adjust the initial amount of the first node by setting the value of P_N1 to a non-zero value.*
- Switch Between Components – Use the drop-down list in the settings table to navigate among nodes, proteases, regulations, and the cell volume. *Note: in the current version of the app, cell volume changes will not affect simulations, as the app considers absolute abundance of molecular species and not their concentration.*
- Save Parameter Changes – Confirm changes by saving before switching tabs.

3. Running Deterministic and Stochastic Simulations

- Set Simulation Species – Under Simulations → Settings, you can add the species you want to track. Example: to follow the protein product of 3 nodes, select P_N_1, P_N_2 and P_N_3.

- Adjust Simulation Time – In Simulation Settings, adjust Stop time. Ensure the simulation window is long enough to capture dynamics. *Example: set Stop time at 1440 minutes to get a simulation over a 24 h period.*
- Run Simulation – Click on Simulation → Run to execute the simulation and visualize the circuit behavior. You can adjust each parameter by selecting it and assigning a new value, this will automatically run a new simulation. Use Clean (or enable Autoclean) to clear previous graphs.
- Modify Solver and Tolerances – Choose the SolverType. The default is *ode15s* for deterministic simulations. You can select a stochastic solver (e.g., Adaptive SSA) if Python, Basico and Copasi are properly installed. Adjust MaxWallClock (simulation duration), absolute, and relative tolerances. *Note: lower tolerances increase accuracy but may slow down computation.*
- Explore Additional Simulation Features
  - Open in SimBiology: Click Simulation → Open in SimBiology for further analysis.
  - Parameter Scan: Select a parameter, then click Parameter Scan to run multiple simulations across a range (adjust min, max, and divisions). *Note: this feature is useful to identify trends and find the parameter range in which the desired behaviour is observed.* By default, the parameter scan displays the final concentrations from the simulation. However, selecting the "Steady State" option ensures that the steady-state concentrations are shown instead.
  - Export to COPASI: If COPASI is installed, click Simulation → Open in COPASI for further analysis.

4. Generating Code

- Export Code – From Input/Output, click Export Code to generate a MATLAB script that replicates your current circuit. This script can be saved, reused, or modified for further work.
- Add Run and Plot Commands – Click "Add run and plot" to include the simulation execution and graphing commands in your exported script. Make sure your preferred solver is selected before exporting, if not using the default.
- Modify and Rebuild Model – Open the generated code in the MATLAB editor to tweak your circuit (e.g., add nodes or change parameters) and run the Rebuild model command to update the circuit in the app. *Note: this feature allows for fast creation of larger modular networks using the command line, and also allows to include additional customized rules.*

5. Spatial Simulations – This function allows to run spatial simulations that take into account diffusion of species and cell growth

- Open Spatial Simulation Tab – Go to Simulation → Spatial simulation – Opens the panel to configure spatial simulations
- Set Simulation Grid Details – Specify spatial divisions in X and Y, L_y length (grid size), simulation time (T), and number of time steps – Higher spatial/temporal resolution increases simulation accuracy and cost.
- Adjust Plot Frequency and Noise – Define number of plots and initial noise – Noise is useful for cell lawn patterns (e.g., in Turing systems).
- Choose Boundary Conditions – Set edge behavior to pbc (periodic boundary conditions) or no flux.

- Use Preliminary Simulation – Enable Preliminary simulation if initial conditions are unknown – Uses the steady-state of a classic simulation to initialize the spatial simulation.
- Select System Type: Lawn vs. Colony – Choose between lawn of cells (homogeneous) or colony growth (expanding from one cell) – Unlocks colony-specific options if colony is selected.
- Configure Colony Parameters – Set:
  - C_{max}: max cell concentration before entering a state of metabolic inactivity
  - Initial colony height (C_0): height of the 2D Gaussian function representing the inoculum
  - Initial colony width: width of the inoculum
  - Growth rate and cell diffusion: parameters of the Fisher equation to model colony growth
- Set Species Diffusion Behavior — For each species, define:
  - Diffusion coefficient: 0 for intracellular or non-diffusible species, > 0 for diffusible species (e.g. quorum sensing molecules)
  - Diffuses with colony: check for intracellular species, uncheck for extracellular species
  - Make sure to scale diffusion coefficient to match physical units (L_y length)
- Run and Visualize Simulation -– Click Run to start spatial simulation. Outputs concentration map for each selected species and cell concentration across the grid (if simulating a colony).
- Compare to Pseudo-2D Image -– Optionally run a basic Figure 2D plot for quick comparison – Reveals if the spatial simulation captures more detail or pattern types than the pseudo-2D image.
- Export Results -– Use Create Figure to save plots, or Save Data to export simulation results — Save Data becomes available only after running a simulation.

1.5 Available solvers

In this section, we summarize the key properties of the solvers available in our application in Appendix Table S1. When using a stochastic solver from COPASI, noise arises intrinsically due to molecular discreteness and reaction probabilities, following the Chemical Master Equation (CME). Reaction events are sampled probabilistically based on reaction propensities (Gillespie-type noise), without incorporating additional extrinsic noise terms such as additive Wiener processes. However, COPASI also supports additional noise sources, including Langevin-like fluctuations, which can be introduced through rate rules in the COPASI GUI. For further details on stochastic simulations, we refer readers to (Gillespie, 1977; Wilkinson, 2012).

It is important to note that simulating stiff systems, particularly when using stochastic solvers, can be more time-consuming. Additionally, only the MATLAB solvers can be accelerated using the `sbioaccelerate` function through MATLAB's code generation capabilities. In the absence of additional knowledge about the system, we recommend using the `sundials` solver in MATLAB for deterministic simulations and the `Stochastic (Adaptive SSA/T-leap)` solver of COPASI for stochastic simulations, which dynamically transitions between the Gillespie Direct Method and the τ-leap approximation depending on system state. This approach ensures both accuracy and computational efficiency, particularly for larger systems. The application also allows setting a maximum runtime for the solver, which can be particularly useful in stochastic simulations due to the larger simulation time. A brief comparison of simulation times, using the repressilator circuit family as an example, can be found in Appendix Section 2.1.

Appendix Table S1: A list and brief description of the available solvers in the application. The solver types are abbreviated as "det." for deterministic, "stoch." for stochastic, and "hybr." for hybrid solvers, with hybrid solvers combining both deterministic and stochastic methods. For further details, refer to the respective documentation on MATLAB and COPASI.

Solver name	Origin	Type	Description
ode15s	MATLAB	det.	A variable-step, variable-order solver designed for stiff systems of differential equations – ideal when the model involves fast and slow dynamics.
ode45	MATLAB	det.	A versatile, medium-accuracy, non-stiff solver using the Runge-Kutta method – best for smooth, moderately complex systems.
sundials	MATLAB	det.	A suite of solvers optimized for large-scale, stiff, or sparse models – useful for complex biological networks and systems biology simulations.
Deterministic (LSODA)	COPASI	det.	Adaptive solver that switches between stiff and non-stiff methods automatically — ideal for models with mixed dynamics.
Deterministic (RADAU5)	COPASI	det.	Implicit Runge-Kutta solver for stiff systems — suitable for highly stable, precise simulations.
Stochastic (Adaptive SSA/T-leap)	COPASI	stoch.	Hybrid stochastic solver that switches between SSA and T-leap based on system dynamics — balances accuracy and speed.
Stochastic (Gibson + Bruck)	COPASI	stoch.	Optimized stochastic simulation algorithm (SSA) that reduces overhead — faster for large reaction networks.
Stochastic (Direct method)	COPASI	stoch.	Classic Gillespie SSA — exact but slower for large systems with frequent reactions.
Stochastic (T-leap)	COPASI	stoch.	Tau-leaping method — approximates many reactions per time step, improving speed for large systems with small fluctuations.
Hybrid (Runge-Kutta)	COPASI	hybr.	Combines stochastic SSA with Runge-Kutta for deterministic parts — ideal for systems with fast and slow species.
Hybrid (LSODA)	COPASI	hybr.	Hybrid solver using LSODA for deterministic components and SSA for stochastic ones — adaptable to stiff, mixed systems.
Hybrid (RK-45)	COPASI	hybr.	Hybrid approach combining SSA with high-order Runge-Kutta (RK-45) for smoother deterministic parts — suitable for complex hybrid models.
SDE Solver (RI5)	COPASI	stoch.	Stochastic Differential Equation (SDE) solver using the RI5 method — handles noise in continuous systems, useful for biochemical models with intrinsic fluctuations.

2 Simulations

2.1 Simulation time

In Appendix Figure S3 and Appendix Table S2, we summarize how simulation time depends on the chosen solvers. While it is important to note that this dependence varies with the system (e.g., stiffer differential equations may show a greater advantage for stiff solvers), as a general trend, we observe that MATLAB solvers perform well, especially when using the `sbioaccelerate` function. Additionally, the `lsoda` solver from COPASI is also quite efficient, whereas stochastic simulations tend to be more time-consuming.

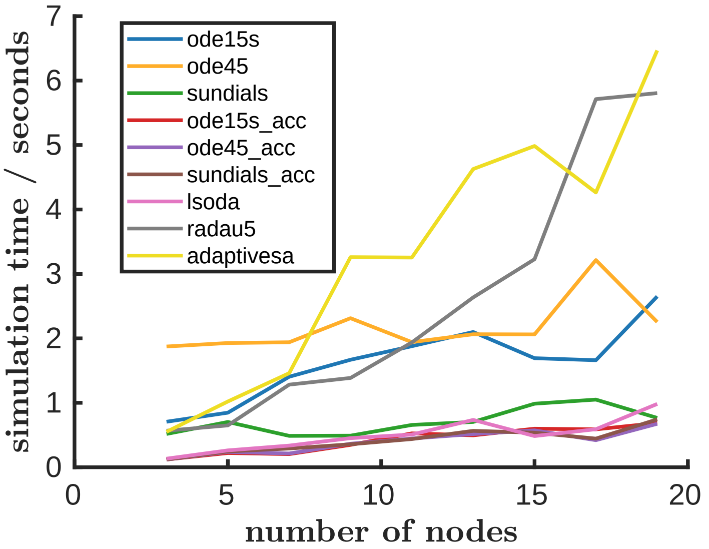

Appendix Figure S3: Simulation time as a function of the number of nodes for the repressilator circuit, using different solvers. The simulation settings are consistent with those in Figure 5e for the repressilator. The relative tolerance was set to 10^{-6} and the absolute tolerance was set to 10^{-8}, while the simulation time was 10^5 minutes. In the legend "acc" refers to the acceleration achieved using the `sbioaccelerate` function. Simulation times were measured using MATLAB's `timeit` function. All simulations were performed on a laptop equipped with a 12th Gen Intel(R) Core(TM) i7-1270P processor, MATLAB R2023b, and running Linux Mint 21.3. The data from the graph are presented in Appendix Table S2.

Appendix Table S2: Simulation time (in seconds) as a function of the number of nodes in a repressilator circuit and the applied solver. The data shown here is the same as in Appendix Figure S3, with a more detailed explanation provided in the figure caption.

#nodes	ode15s	ode45	sundials	ode15s_acc	ode45_acc	sundials_acc	lsoda	radau5	adaptivesa
3	0.7063	1.8737	0.5168	0.1208	0.1194	0.1207	0.1331	0.5669	0.5502
5	0.8468	1.9277	0.7025	0.2212	0.2385	0.2459	0.2619	0.6493	1.0204
7	1.4045	1.9396	0.4872	0.2042	0.2135	0.2909	0.3361	1.2819	1.4598
9	1.6680	2.3128	0.4903	0.3473	0.3650	0.3564	0.4518	1.3857	3.2599
11	1.8785	1.9416	0.6567	0.5258	0.4456	0.4376	0.5079	1.9369	3.2543
13	2.0987	2.0641	0.7034	0.4956	0.5150	0.5649	0.7345	2.6356	4.6274
15	1.6923	2.0614	0.9861	0.5983	0.5722	0.5364	0.4843	3.2280	4.9847
17	1.6610	3.2145	1.0498	0.5884	0.4203	0.4443	0.5902	5.7122	4.2639
19	2.6507	2.2536	0.7657	0.6860	0.6745	0.7426	0.9824	5.8059	6.4679

2.2 Elowitz-model

The specifics of the Elowitz model are provided in Appendix Table S3. In reaction R_1, the ith node generates mRNA, which can be inhibited by a transcription factor originating from the jth node. Reaction R_2 describes the production of the transcription factor associated with the ith node.

Appendix Table S3: The Elowitz-type transcription factor model (Elowitz & Leibler, 2000) describes the production of mRNA and protein ($mRNA_i$, P_i) at the ith node, which is repressed by another transcription factor, P_j. This repression is modeled using a Hill function: $HILL([P_j]) = \frac{1}{1+([P_j]/K)^n}$, where $K = 40$ molecule and $n = 2$.

Nr.	Reaction	Rate law	Rate constant (Elowitz & Leibler, 2000)	Unit
R_1	$\emptyset \longleftrightarrow mRNA_i$	$r_1 = k_0 + k_1 \cdot HILL([P_j])$	$k_0 = 0.03$	molecule/minute
			$k_1 = 30$	molecule/minute
		$r_{1r} = k_2\,[mRNA_i]$	$k_2 = 0.3466$	1/ minute
R_2	$\emptyset \longleftrightarrow P_i$	$r_2 = k_3\,[mRNA_i]$	$k_3 = 6.9315$	1/ minute
		$r_{2r} = k_4\,[P_i]$	$k_4 = 0.0693$	1/ minute

Here we showcase how to create a model for the repressilator with our tool using the command line functionalities:

Listing 1: Building the model for the Repressilator

```
Ecoli = Cell('Elowitz');
Ecoli = Ecoli.add_node('N1','type1');
Ecoli = Ecoli.add_node('N2','type1');
Ecoli = Ecoli.add_node('N3','type1');
Ecoli = Ecoli.add_regulator('Repression_in','N2','HILL','P_N1');
Ecoli = Ecoli.add_regulator('Repression_in','N3','HILL','P_N2');
```

```
Ecoli = Ecoli.add_regulator('Repression_in','N1','HILL','P_N3');
```

2.3 Tomazou-model

In Appendix Table S4 we present the Tomazou-type transcription factor model (Tomazou et al, 2018). The mRNA production of the ith node can be repressed by the transcription factor of the jth node, P_j. This repression is described with the following Hill-function: $\mathrm{HILL}([P_j]) = \frac{1}{1+([P_j]/K)^n}$, where $K = 5$ molecule and $n = 2$. When external inducers are present (I_1 and I_2 in Figure 3II-a,b in the manuscript, $[P_j]$ can be replaced with the concentration of the inducers in the Hill-function and $K = 50$ μM. The protease degradation rate is determined using Michaelis-Menten kinetics: $k_{\mathrm{protease}} = \frac{k_{\mathrm{protease,max}}[\mathrm{PROT}]}{K_{\mathrm{protease}} + \mathrm{Substrates}}$, where $k_{\mathrm{protease,max}} = 50$ 1/ minute, $K_{\mathrm{protease}} = 30$ molecule and "Substrates" represents the sum of all proteins degraded by the given protease ($\mathrm{Substrates} = \sum_i [P_i]$).

Appendix Table S4: The Tomazou-type transcription factor model (Tomazou et al, 2018). Further explanation can be found in the text.

Nr.	Reaction	Rate law	Rate constant (Tomazou et al, 2018)	Unit
R_1	$\emptyset \longleftrightarrow \mathrm{mRNA}_i$	$r_1 = n_{\mathrm{copy}}(a_0 + a_1 \cdot \mathrm{HILL}([P_j]))$	$n_{\mathrm{copy}} = 25$	molecule
			$a_0 = 0.001$	1/ minute
			$a_1 = 100$	1/ minute
		$r_{1r} = (k_{\mathrm{mRNA,degr}} + k_d)\,[\mathrm{mRNA}_i]$	$k_{\mathrm{mRNA,degr}} = 0.5$	1/ minute
R_2	$\emptyset \longleftrightarrow \mathrm{uP}_i$	$r_2 = k_{\mathrm{translation}}\,[\mathrm{mRNA}_i]$	$k_{\mathrm{translation}} = 6.9315$	1/ minute
		$r_{2r} = (k_d + k_{\mathrm{protease,i}})\,[\mathrm{uP}_i]$	$k_d = 0.01$	1/minute
R_3	$\mathrm{uP}_i \longrightarrow P_i$	$r_3 = k_{\mathrm{mat}}\,[\mathrm{uP}_i]$	$k_{\mathrm{mat}} = 0.4$	1/ minute
R_4	$P_i \longrightarrow \emptyset$	$r_4 = (k_d + k_{\mathrm{protease,i}})\,[P_i]$		

Here we showcase how to create the model presented in Figure 3II of the manuscript:

Listing 2: Building the model for independent amplitude and frequency modulation in a re-designed Repressilator

```
%% Add nodes
Ecoli = Ecoli.add_node('R1');
Ecoli = Ecoli.add_node('R2');
Ecoli = Ecoli.add_node('R3');
Ecoli = Ecoli.add_node('G');

%% Add protease
Ecoli = Ecoli.add_protease('R1','C');
Ecoli = Ecoli.add_protease('R2','C');
Ecoli = Ecoli.add_protease('R3','C');
Ecoli = Ecoli.add_protease('G','L');

%% Add regulators
Ecoli = Ecoli.add_regulator('Repression_in','R1','R3');
Ecoli = Ecoli.add_regulator('Repression_in','R2','R1');
Ecoli = Ecoli.add_regulator('Repression_in','R3','R2');
```

```
Ecoli = Ecoli.add_regulator('Repression_in','G','R3');
Ecoli = Ecoli.add_regulator('Activation_in','R2','Y');
Ecoli.set('Y','Constant',true,'R2','Y');
Ecoli = Ecoli.add_regulator('Activation_out','R2','Y','I2');
Ecoli = Ecoli.add_regulator('Activation_in','G','U');
Ecoli.set('U','Constant',true,'G','U')
Ecoli = Ecoli.add_regulator('Activation_out','G','U','I1');
```

2.4 CRISPR model

To achieve comparable concentrations between the Santos-Moreno model (Santos-Moreno et al, 2023) and the Elowitz model, we fine-tuned several parameters, as detailed in Appendix Table S5 of the manuscript. Since in the Santos-Moreno model, the dilution rate k_d is accounted for separately, we modified the original reaction constants for the degradation of proteins and RNAs by incorporating this parameter: $d_P = 0.0693 - k_d$ and $d_{RNA} = 0.3466 - k_d$. Additionally, the rate law for R_1 was slightly adjusted from the original Elowitz model to explicitly consider the DNA concentration ($[\mathrm{DNA}]_0 =$ 30 molecules), resulting in the parameter correction: $a_1 = 30/30 = 1$ minute^{-1}.

RNA production was scaled by a factor of $r = 22.7$ using the Elowitz parameters for R_1–R_3, and we increased the production rates in R_4 and R_6 (k_{fds}, k_{fdsd}) with this factor as well. The Santos-Moreno model introduced additional algebraic equations to maintain overall concentrations of dCas and DNA, leading to differential-algebraic equations (DAE). In our modified model, we adjusted R_5 and R_6 to produce these species during dilution, thus ensuring mass conservation for DNA and dCas and converting the system to ordinary differential equations (ODE).

Appendix Table S5: Modified model for the CRISPRlator. Initial concentrations are set at $[\mathrm{DNA}]_0 = 30$ molecules and $[\mathrm{dCas}]_0 = 1434$ molecules (Santos-Moreno et al, 2023). All rate constants are provided in units of *minute* and *molecule*. The repression of the jth node on the ith node occurs through the formation of the DNA$_j$-sgRNA$_i$ complex, as described in the R_6 reaction.

Nr.	Reaction	Rate law	Rate constant	Ref.
R_1	$\emptyset \longleftrightarrow \mathrm{mRNA}_i$	$r_1 = a_0 + a_1\,[\mathrm{DNA}_i]$	$a_0 = 0.03$	(Elowitz & Leibler, 2000)
		$r_{1r} = (k_d + d_{\mathrm{RNA}})[\mathrm{mRNA}_i]$	$a_1 = 1$	
R_2	$\emptyset \longleftrightarrow \mathrm{sgRNA}_i$	$r_2 = a_0 + a_1\,[\mathrm{DNA}_i]$	$d_{\mathrm{RNA}} = 0.3286$	(Elowitz & Leibler, 2000)
		$r_{2r} = (k_d + d_{\mathrm{RNA}})[\mathrm{sgRNA}_i]$	$k_d = 0.018$	
R_3	$\emptyset \longleftrightarrow P_i$	$r_3 = k_P\,[\mathrm{mRNA}_i]$	$k_P = 6.9315$	(Elowitz & Leibler, 2000)
		$r_{3r} = (k_d + d_P)[P_i]$	$d_P = 0.0513$	
R_4	$\mathrm{dCas} + \mathrm{sgRNA}_i \longleftrightarrow \mathrm{dCas{:}sgRNA}_i$	$r_4 = k_{\mathrm{fds}}[\mathrm{dCas}][\mathrm{sgRNA}_i]$	$k_{\mathrm{fds}} = 1.4674$	(Santos-Moreno et al, 2023)
		$r_{4r} = k_{\mathrm{rds}}[\mathrm{dCas{:}sgRNA}_i]$	$k_{\mathrm{rds}} = 0.0776$	
R_5	$\mathrm{dCas{:}sgRNA}_i \longrightarrow \mathrm{dCas}$	$r_5 = k_d[\mathrm{dCas{:}sgRNA}_i]$		(Santos-Moreno et al, 2023)
R_6	$\mathrm{dCas{:}sgRNA}_i + \mathrm{DNA}_j \longleftrightarrow \mathrm{dCas{:}sgRNA}_i\mathrm{:DNA}_j$	$r_6 = k_{\mathrm{fdsd}}[\mathrm{dCas{:}sgRNA}_i][\mathrm{DNA}_j]$	$k_{\mathrm{fdsd}} = 0.2670$	(Santos-Moreno et al, 2023)
		$r_{6r} = k_{\mathrm{rdsd}}[\mathrm{dCas{:}sgRNA}_i\mathrm{:DNA}_j]$	$k_{\mathrm{rdsd}} = 0$	
R_7	$\mathrm{dCas{:}sgRNA}_i\mathrm{:DNA}_j \longrightarrow \mathrm{dCas} + \mathrm{DNA}_j$	$r_7 = k_d[\mathrm{dCas{:}sgRNA}_i\mathrm{:DNA}_j]$		(Santos-Moreno et al, 2023)

2.5 The coherent and incoherent feed-forward loop

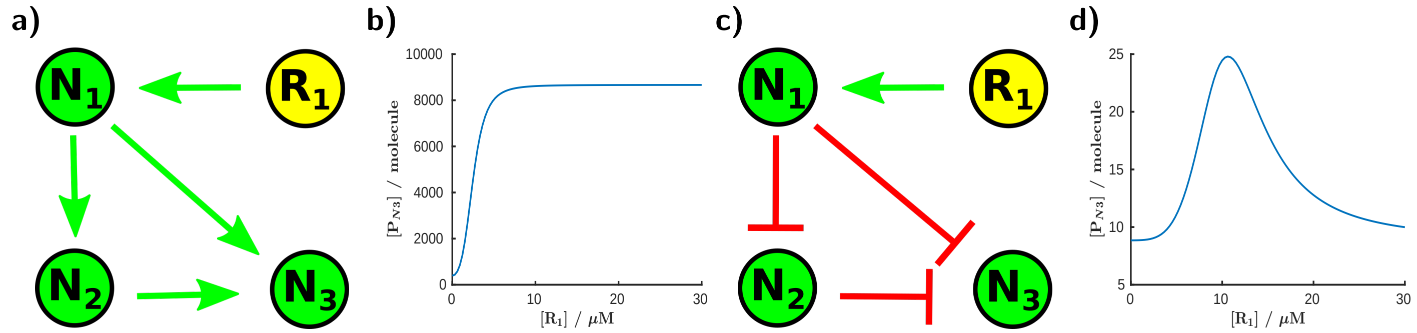

Appendix Figure S4: The behavior of coherent and incoherent feed-forward loops (CFFL and IFFL) with three nodes: N_1, N_2, and N_3, where R_1 serves as the inducer. The protein concentration produced by the third node, P_{N3}, is calculated as a function of the inducer concentration. The graphs display: (a) the circuit of a CFFL, (b) the output protein concentration as a function of the inducer concentration, (c) the circuit of an IFFL, and (d) the output protein concentration as a function of the inducer concentration. The detailed information about the models are available in the files "CFFL.html" and "IFFL.html".

Listing 3: Building the model for a coherent feed-forward loop and an incoherent feed-forward loop:

```
%% coherent feed-forward loop
Ecoli = Ecoli.add_node('N1','type1');
Ecoli = Ecoli.add_node('N2','type1');
Ecoli = Ecoli.add_node('N3','type1');
Ecoli = Ecoli.add_regulator('Activation_in','N2','HILL','P_N1');
Ecoli = Ecoli.add_regulator('Activation_in','N3','HILL','P_N2');
Ecoli = Ecoli.add_regulator('Activation_in','N3','HILL','P_N1');
Ecoli = Ecoli.add_regulator('Activation_out','N1','HILL','R1');

%% incoherent feed-forward loop
Ecoli = Ecoli.add_node('N1','type1');
Ecoli = Ecoli.add_node('N2','type1');
Ecoli = Ecoli.add_node('N3','type1');
Ecoli = Ecoli.add_regulator('Activation_out','N1','HILL','R1');
Ecoli = Ecoli.add_regulator('Repression_in','N2','HILL','P_N1');
Ecoli = Ecoli.add_regulator('Repression_in','N3','HILL','P_N1');
Ecoli = Ecoli.add_regulator('Repression_in','N3','HILL','P_N2');
```

2.6 Implementing systems with self-regulating nodes

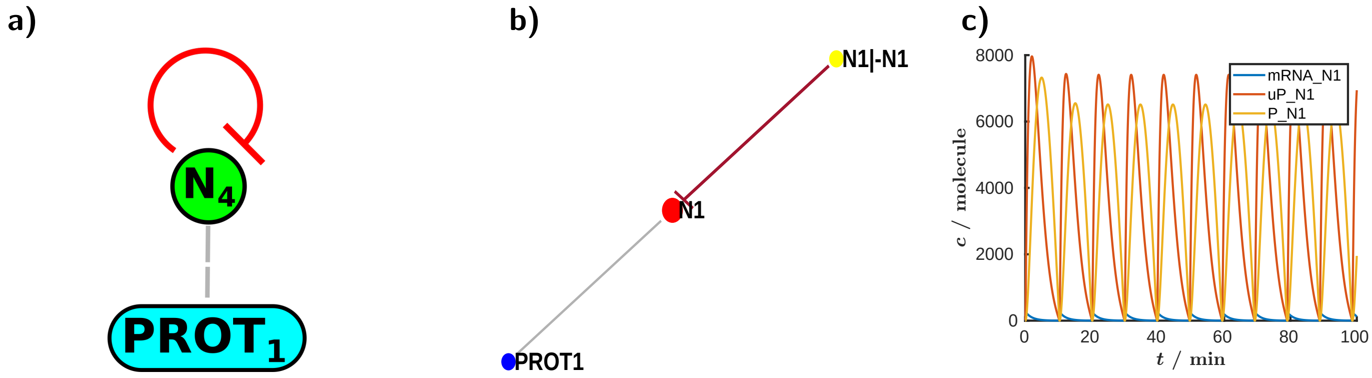

Appendix Figure S5: Representation of self-regulating nodes in GRN_modeler, demonstrated using the Goodwin oscillator (Goodwin, 1963). This circuit includes a self-repressing node, modeled with the Tomazou framework to account for protease system effects. (a) The Goodwin oscillator circuit. (b) Visualization of the self-repressing node in GRN_modeler. (c) Time dynamics of mRNA ($mRNA_{N1}$), unfolded protein (uP_{N1}), and protein concentration (P_{N1}). Detailed model information is provided in "Goodwin.html".

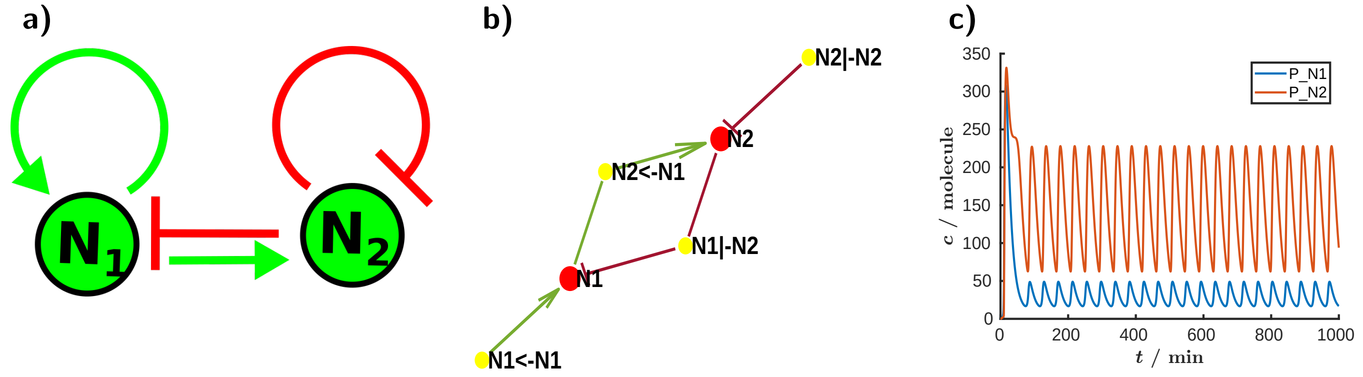

Appendix Figure S6: Visualization of self-regulating nodes in GRN_modeler, demonstrated with the Stricker oscillator (Stricker et al, 2008). (a) The Stricker oscillator circuit. (b) Representation of a self-repressing and self-activating node in GRN_modeler. (c) Time dynamics of the protein concentrations (P_{N1}, P_{N2}). The model is based on a modified Elowitz framework, with the protein degradation rate set to $d_P = 0.2$ molecules. To induce oscillations, we increased nonlinearity by applying a Hill exponent of 6 for both activation and repression interactions between nodes N_1 and N_2. Further model details are available in"stricker.html".

2.7 Finding steady states in the toggle switch

In Appendix Figure S7, we demonstrate the bistable behavior of the toggle switch as visualized using the GNR_modeler. The mutual repression between nodes ensures that when one node is activated, the other is suppressed, driving this dynamic behavior. Panels (c) and (d) show that deterministic simulations lead the system to one of two stable fixed points, depending on the initial concentrations. In contrast, stochastic simulations reveal that small differences in initial

conditions are insufficient to determine the system's outcome. However, larger initial differences produce behavior similar to the deterministic results (Appendix Figure S7f).

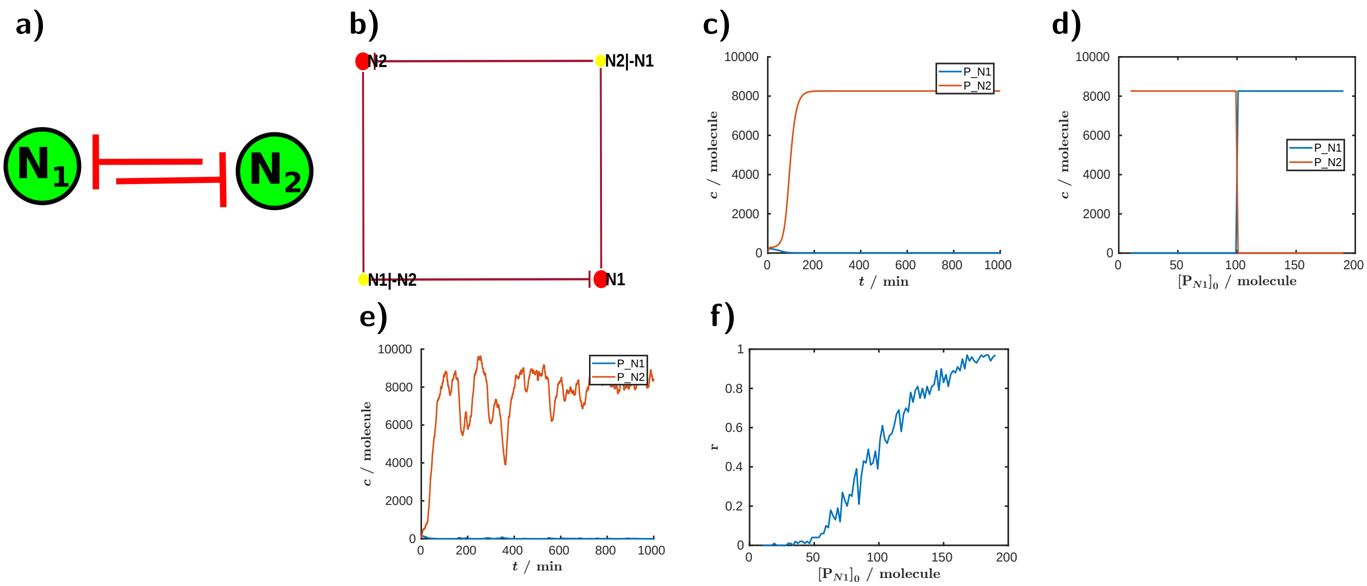

Appendix Figure S7: Simulation of the toggle switch using the Elowitz model. (a) Circuit topology. (b) Visualization of the circuit within the application. (c) Time evolution of protein concentrations when the initial concentration of P_{N2} exceeds that of P_{N1} ($[P_{N2}]_0 = 100$ molecules, $[P_{N1}]_0 = 90$ molecules), simulated using MATLAB's deterministic "ode15s" solver. (d) Steady-state concentrations as a function of the initial P_{N1} concentration. The initial P_{N2} concentration was fixed at 100 molecules in all simulations. (e) An example stochastic simulation performed with COPASI's "adaptivesa" solver, using the same setup as the deterministic simulation in panel (c). (f) The ratio (r) of cases where $[P_{N1}] > [P_{N2}]$ after 100 stochastic simulations for each initial concentration. The initial P_{N2} concentration was fixed at 100 molecules in all simulations. Detailed model information is provided in "Toggle.html."

2.8 Novel oscillator circuits

2.8.1 The "actolator" family

In the case of the repressilator, even a single-node circuit can oscillate, albeit through a different mechanism. The one-node oscillator is known as the Goodwin oscillator (Goodwin, 1963). Oscillations can arise from high non-linearity in the system and inherent delays (Gonze & Ruoff, 2021), or from a positive feedback loop with lower non-linearity (Ananthasubramaniam & Herzel, 2014). Similarly, the actolator can exhibit oscillations with a two-node circuit, but achieving this requires a higher degree of non-linearity, typically a Hill exponent of at least 6, while generally used Hill exponents are around 2 (see the Elowitz-model in Appendix Table S3 and Appendix Figure S8).

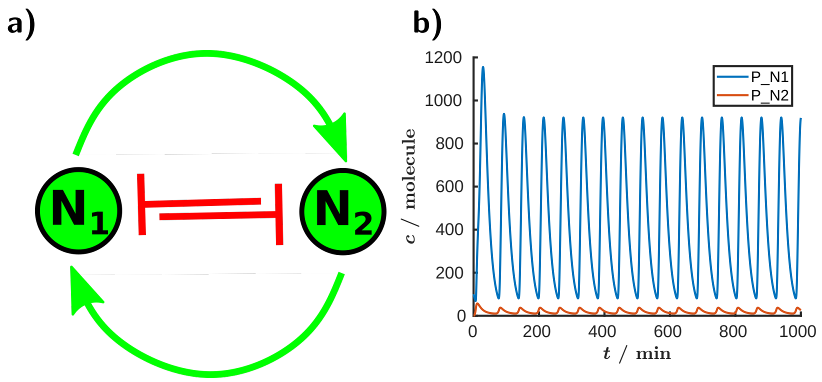

Appendix Figure S8: The "actolator" with a 2-node circuit. While the circuits in Figure 5 with 4, 6, 8, and 12 nodes exhibit oscillatory behavior with the original parameter set, the 2-node circuit required higher-order nonlinearity to induce oscillations. a) Circuit topology. b) Protein oscillations for the first (blue) and second node (red). Detailed information about the models is available in the file "actolator2.html".

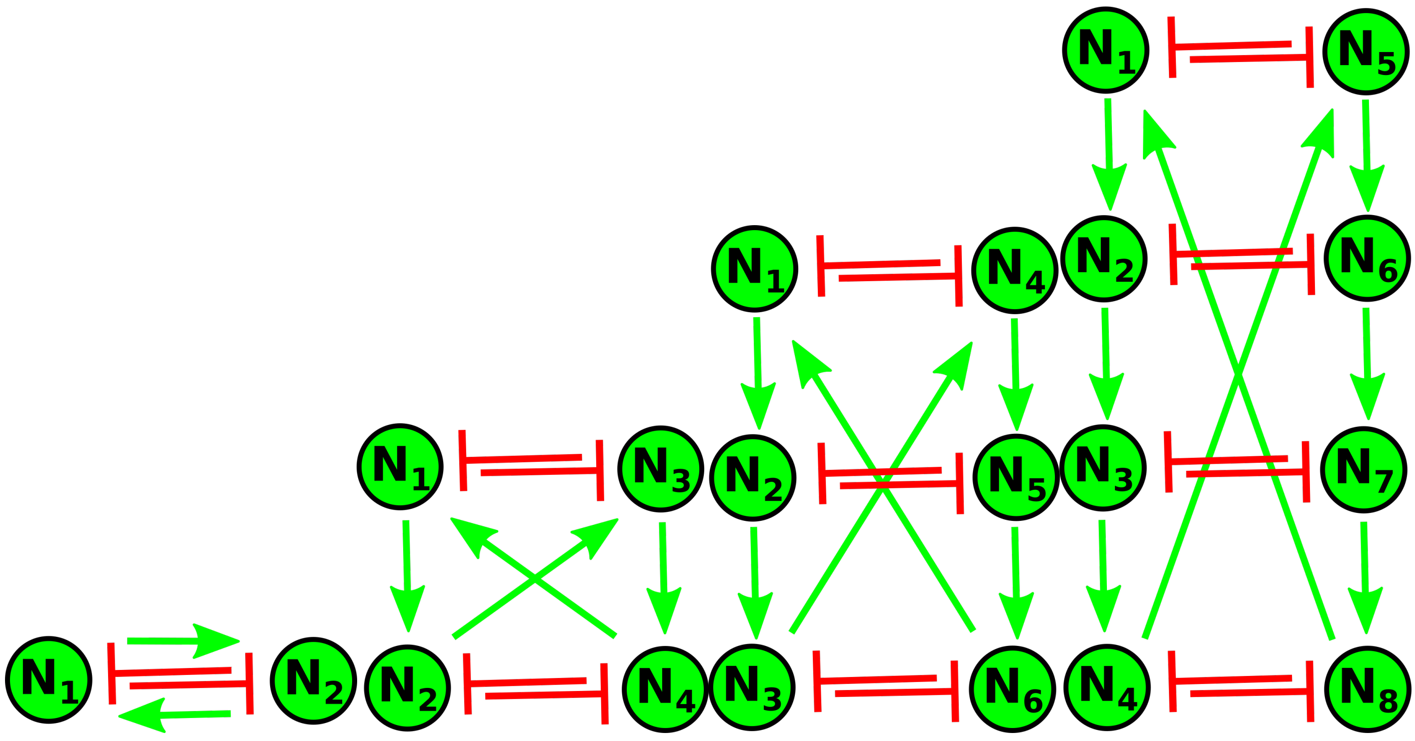

Appendix Figure S9: Alternative representation for the actolator family to highlight the role of the toggle switches. We show the 2, 4, 6, 8 node actolator respectively. In the case of the two node circuit, higher non-linearity is necessary for oscillation.

2.8.2 Modified repressilator circuits: the "acrelator" family

In Appendix Figure S10 we demonstrate that when n repressive interactions are replaced in an N-node circuit with activations, its oscillatory behavior resembles that of a repressilator with $N - n$ "effective nodes". If the number of "effective nodes" is odd, the circuit may exhibit oscillatory behavior. However, in many cases, achieving this requires a higher non-linearity, meaning a larger Hill exponent (n_{Hill}). For example, for a "x-y" circuit (Appendix Figure S12), $n_{Hill} \geq 6$ is needed, which is experimentally difficult to achieve, suggesting that such circuits may not oscillate in reality (Hodgkin & Huxley, 1952). For a "4-1" circuit (Appendix Figure S11a), with a $n_{Hill} = 2$, the system exhibits damped oscillations, indicating that the fixed point is a stable focus. To achieve sustained oscillations, $n_{Hill} = 3$ was sufficient in this case, suggesting that this configuration might be experimentally feasible. We can observe a similar situation in the 6-node circuit: replacing one repressive interaction with activation enables oscillations with $n_{Hill} = 3$, while the "6-3" symmetrical circuit can oscillate even with the standard Hill exponent ($n_{Hill} = 2$). The "4-3" circuit, which includes one repression and three activation interactions, serves as a fundamental architecture for action potential oscillations. Indeed, this kind of circuit is not new to the oscillators field, and has been proposed to model the interactions between polarization, Na^+ flux, depolarization, and K^+ flux (Li & Yang, 2018; Hodgkin & Huxley, 1952).

It is worth discussing some additional interesting properties of these circuits, specifically the order of the nodes and their duty cycles. Due to the low half-saturation constants compared to the maximal protein concentrations, a node will activate almost simultaneously with its activating partner. Appendix Figure S12 illustrates this coordinated activation with the "4-3" circuit, where each node is activated nearly simultaneously. In a repressilator, the activation order of the nodes is straightforward: the next node to activate is the second one in the sequence, as two repressive interactions lead to one activation. When activations are also present in the circuit, they can be combined into a single node, causing these nodes to activate simultaneously. After this, the activation order can be determined using the same rules applied in the repressilator. However, we can observe notable differences in the duty cycles of the nodes. For example, in an activation chain like that in the "4-3" circuit, the first node in the chain will deactivate first, with subsequent nodes deactivating only afterward due to the low half-saturation constants. Consequently, the duty cycle will be longer for nodes positioned later in the chain, creating an asymmetry in the duty cycle that may be beneficial for certain applications. If a short duty cycle is desired during oscillations, selecting a node that is repressed by other nodes might be optimal. Conversely, for a node with a longer activation period, one connected through activation interactions would be preferable. Furthermore, nodes with a shorter duty cycle reduce the cellular burden.

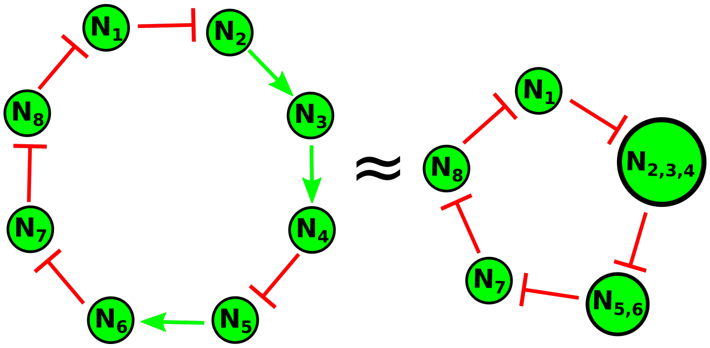

Appendix Figure S10: The "8-3 acrelator". Schematic representation of how oscillatory circuits with an even number of nodes can be created using only consecutive repressive or activating interactions, minimizing the number of regulatory steps. In this example, the eight-node circuit contains three activations, effectively reducing it to a five-node repressilator, which can exhibit oscillatory behavior. For simplicity, we refer to this circuit as the "8-3" circuit in the text. The different size of the nodes in the equivalent repressilator represents the asymmetry in the circuit, while multiple numbers in nodes – connected by activation – indicate that these nodes function synchronously. Further simulations of these types of circuits can be found in Figures Appendix Figure S11 and Appendix Figure S12.

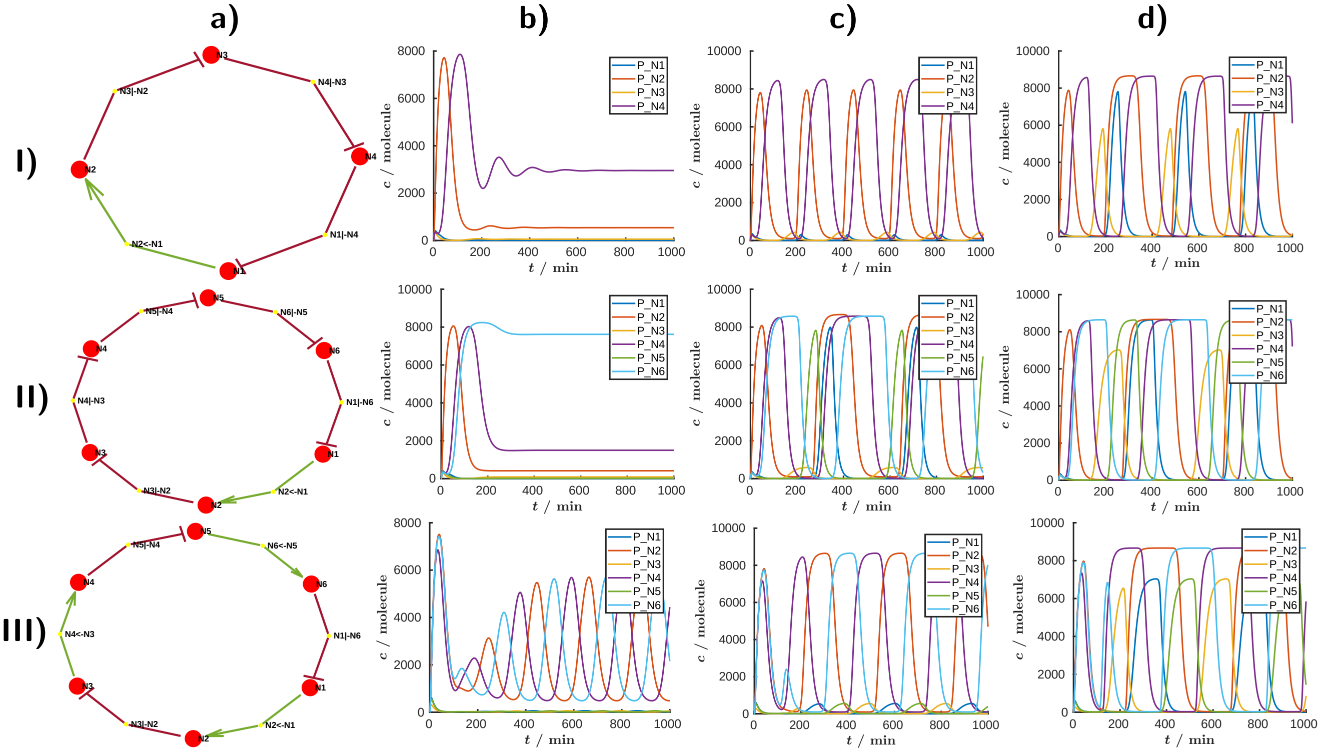

Appendix Figure S11: Examples of the "acrelator" family. These circuits are based on the repressilator family with an even number of nodes, where an odd number of edges have been replaced by activation interactions. I) A four-node circuit with one activation edge, II) a six-node circuit with one activation edge, and III) a six-node circuit with alternating repression and activation edges. a) Circuit topology, b), c), d) trajectories showing the protein concentrations over time for Hill exponents of 2, 3, and 4, respectively. Detailed model information is provided in the files "N4_A1.html", "N6_-A1.html", and "N6_A135.html". Simulations were conducted using the repressilator model from (Elowitz & Leibler, 2000).

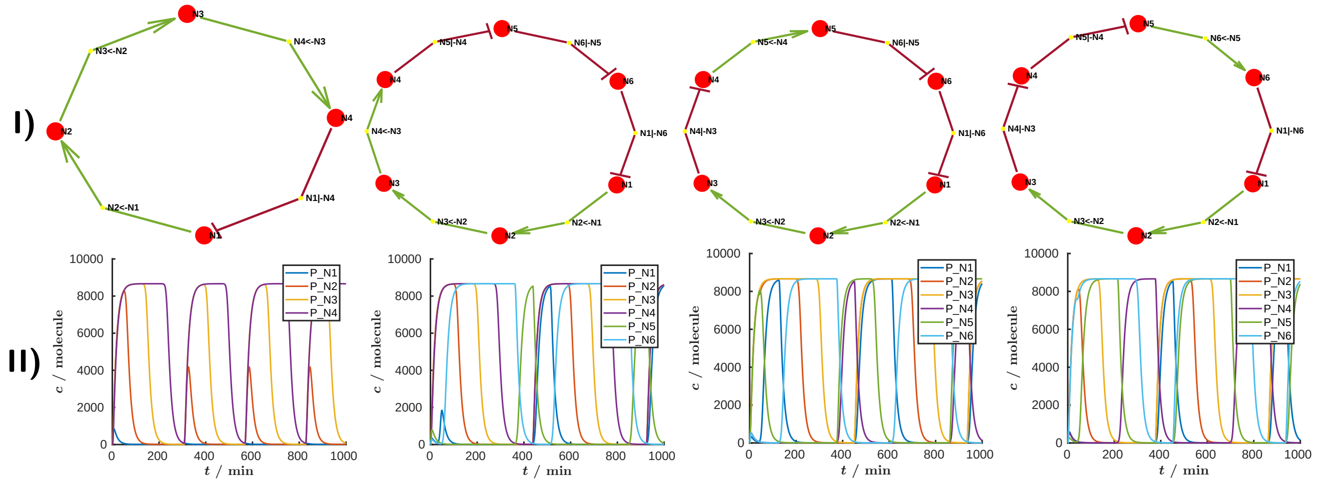

Appendix Figure S12: Examples of the "acrelator" family. These are based on the repressilator family with an even number of nodes, where an odd number of edges have been replaced by activation interactions. These circuits in this figure oscillate only with a higher Hill exponent, which was set to 6 for these simulations. I) Circuit topology, and II) the corresponding trajectories, showing protein concentrations over time. Detailed model information is available in the files "N4_A123.html", "N6_A123.html", "N6_A124.html", and "N6_A125.html". Simulations were performed using the repressilator model from (Elowitz & Leibler, 2000).

2.9 Light biosensor

Appendix Table S6: Fitted parameters for the light system in Eq. 1 to the experimental data shown Figure 7a. The fitting was performed using MATLAB's `lsqnonlin` function, with a function tolerance of 10^{-14} and a termination tolerance of 10^{-10} for the independent variable. All parameters were fitted in logarithmic form, except for the Hill exponents.

Parameter	Value	Unit	Parameter	Value	Unit
k_{light}	0.3185	dimensionless			
K_{light}	156.9	%	K_{ara}	$2.756{\cdot}10^{-4}$	%
n_{light}	0.9897	dimensionless	n_{ara}	1.1177	dimensionless

To implement double-input regulations, new regulation types must be integrated into the node models. We illustrated this process using this example in Appendix Figure S13. Looking ahead, we also plan to develop a dedicated application to support the node model construction process.

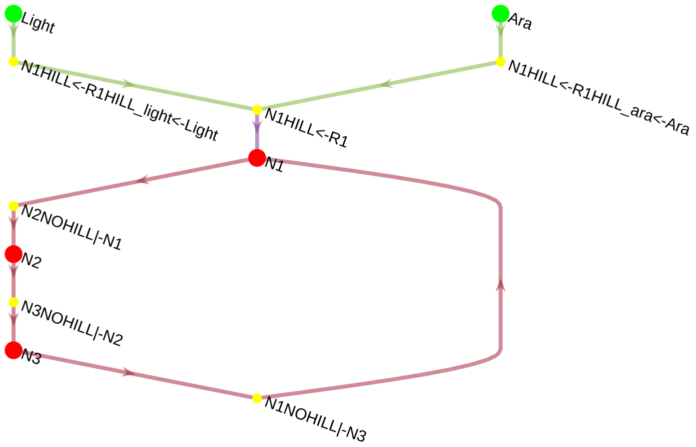

Appendix Figure S13: The three-node CRISPRlator circuit has two inputs: light and arabinose (Ara). The three nodes, N1, N2, and N3, repress each other consecutively through CRISPR interactions, with this input named “NOHILL”. The N1 node is activated by N1HILL<-R1, which is activated by both light (N1HILL<-R1HILL_light<-Light) and arabinose (N1HILL<-R1HILL_ara<-Ara). This figure was created using the `make_graph()` method of the application (with a layered layout) and serves as an example of how to create multiple inputs for a node.

3 Experiments

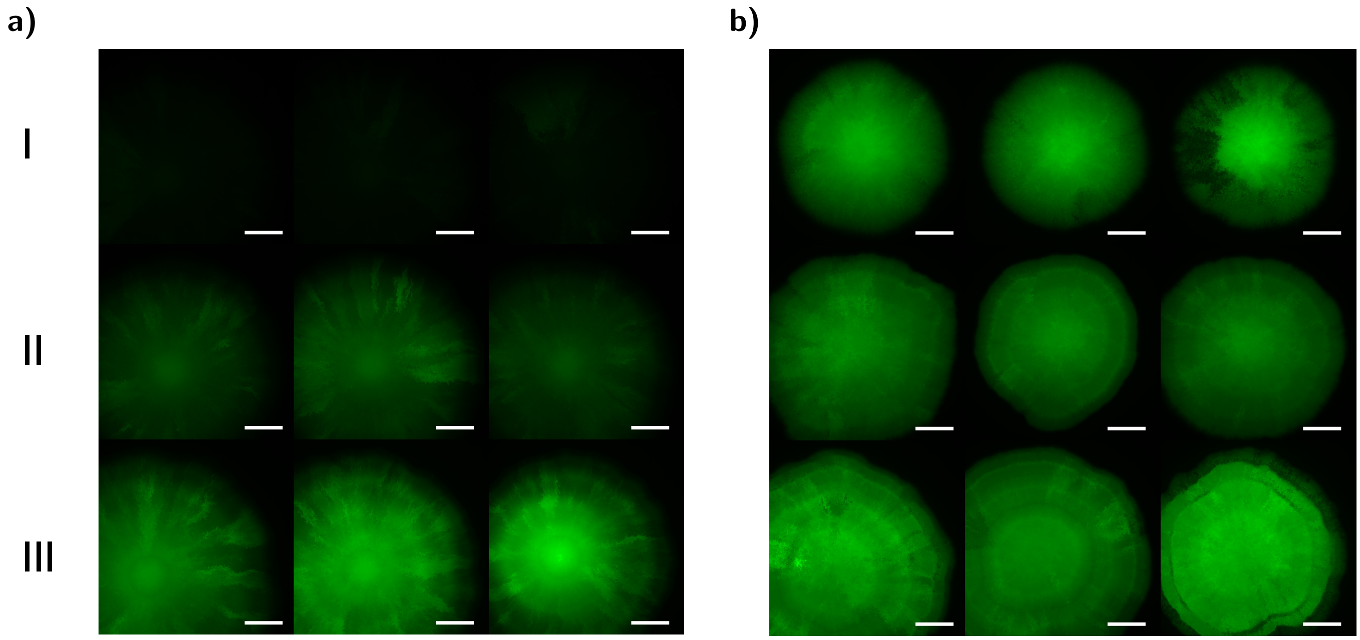

Appendix Figure S14: Fluorescence microscopy images of colonies harboring the light-inducible 1-node circuit (pJP_1node and pJP_Bla01). Colonies grew for 4 days under square wave light pulses (T = 24h) and 50% duty cycle. a - Cultures without L-arabinose. b - Cultures with 0.2% L-arabinose. I. No light exposure, II - light pulses with 25% of the maximal intensity of the LITOS device, III - light pulses with 100% of the maximal intensity of the LITOS device. Scale bars = 1 mm.

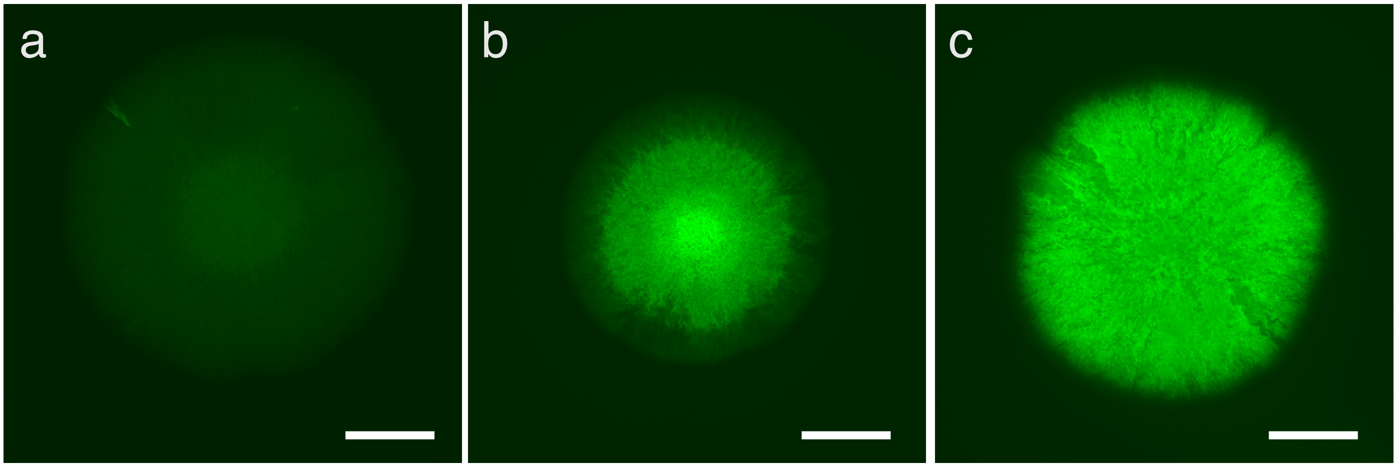

Appendix Figure S15: The light-inducible CRISPRlator (pJP_Osc05, pJP_Bla01 and pJ1996_v2) is not oscillating on solid surface. Fluorescence microscopy pictures of colonies grown for 5 days in dark condition (a), at constant light of 40% intensity (b) and in the dark with 0.2% L-arabinose (c). Scale bars = 1 mm.

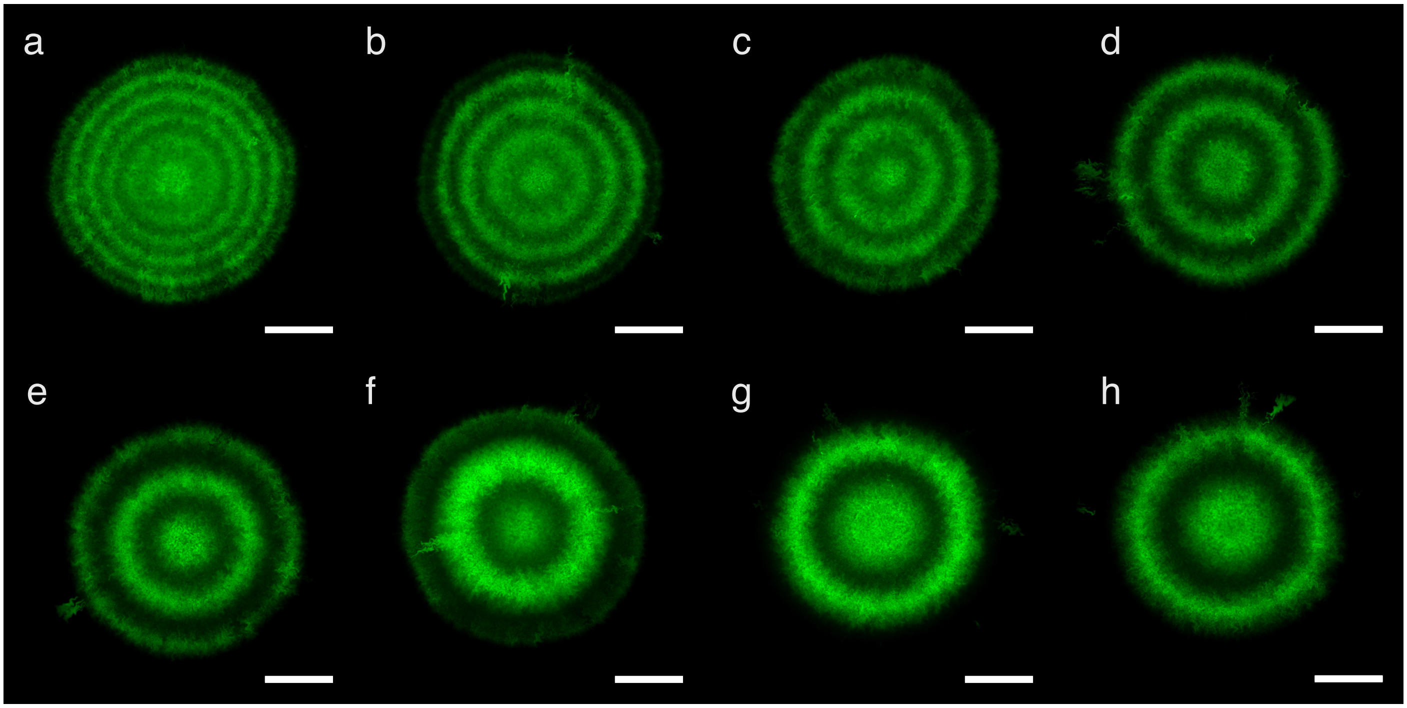

Appendix Figure S16: The number of rings generated by the light-inducible CRISPRlator matches with the number of light pulses. Fluorescence microscopy pictures of colonies harboring the light-inducible CRISPRlator (pJP_Osc05, pJP_-Bla01 and pJ1996_v2). mCitrine is represented in green. The colonies grew for 4 days under square wave light pulses (a - 12h, b - 14h, c - 16h, d - 18h, e - 20h, f - 22, g - 24h, h - 26h), with a duty-cycle of 50%. Scale bars = 1 mm.

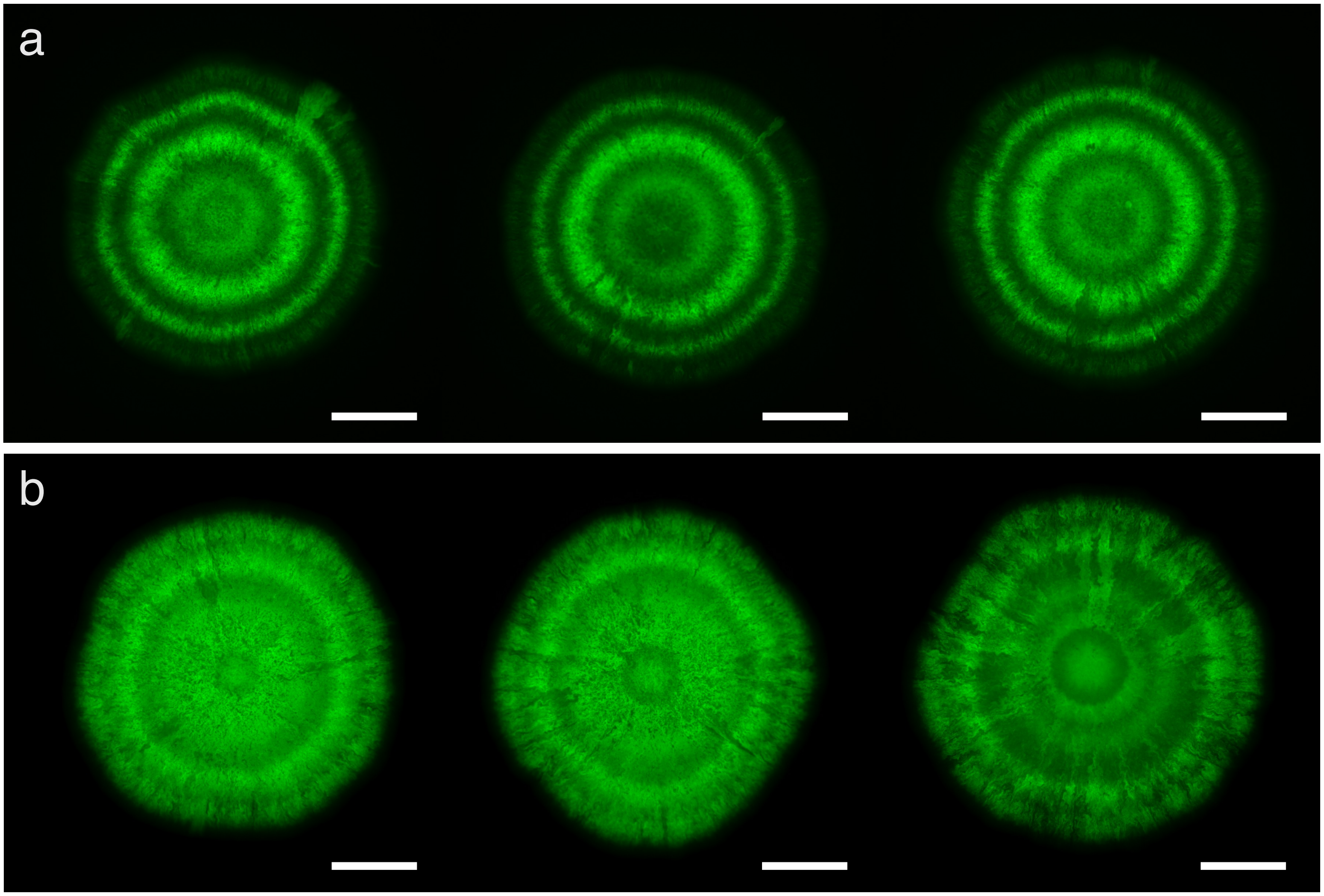

Appendix Figure S17: Phase and anti-phase ring patterns by the light-inducible CRISPRlator (pJP_Osc05, pJP_Bla01 and pJ1996_v2). a) Three replicates of colonies grown on solid surface and subjected to light pulses from 0% to 15% intensity (41.66% duty-cycle) for 4 days. The first and the second images are also shown in Figure 6E and Figure 8D, respectively. b) Colonies grown under the same condition as in (a), but with addition of 0.2% L-arabinose. The second image is the same as shown in Figure 8E. Scale bars = 1 mm.

Plasmid name	Description	Reference Number (Addgene)	Reference
pBLADE_-ONLY_C	Plasmid encoding the light-inducible VVD-AraC.	#168050	(Romano et al, 2021)
pJP_Bla01	Modified pBLADE_ ONLY_C, with gentamicin resistance gene and deleted f1 origin of replication.	#230982	This work
pJ1996_v2	Contains *dCas9* and *csy4* necessary for CRISPRi.	#140664	(Santos-Moreno et al, 2020)
pJ2072 (1-OS2)	L-arabinose inducible CRISPRlator, it is composed of three nodes, with each node producing a sgRNA and a fluorescent reporter. It was used as backbone to construct pJP_Osc05.	–	(Santos-Moreno et al, 2023)
pJP_Osc05	pJ2072 (1-OS2) without *araC* and Kanamycin resistance gene placed between mCitrine and mCerulean nodes.	#229040	This work
pCOLA-AraC-pBad-GFP	colA backbone with pBad promoter controlling the expression of sfGFP.		(Duarte et al, 2017)
pJP_Ctrl04	pCOLA-AraC-pBad-GFP without *araC*.	#230983	This work
pJP_1Node	pJP_Ctrl04 with pBad controlling the expression of mCitrine instead of sfGFP.	#230984	This work

Appendix Table S7: List of plasmids used in this work.

References

Ananthasubramaniam B & Herzel H (2014). “Positive Feedback Promotes Oscillations in Negative Feedback Loops”. *PLOS ONE* 9.8, pp. 1–11.

Cao Y, Ryser MD, Payne S, Li B, Rao CV & You L (2016). “Collective Space-Sensing Coordinates Pattern Scaling in Engineered Bacteria”. *Cell* 165.3, pp. 620–630.

Duarte JM, Barbier I & Schaerli Y (2017). “Bacterial microcolonies in gel beads for high-throughput screening of libraries in synthetic biology”. *ACS synthetic biology* 6.11, pp. 1988–1995.

Elowitz MB & Leibler S (2000). “A synthetic oscillatory network of transcriptional regulators”. *Nature* 403.6767, pp. 335–338.

Gillespie DT (1977). “Exact stochastic simulation of coupled chemical reactions”. *The Journal of Physical Chemistry* 81.25, pp. 2340–2361.

Gonze D & Ruoff P (2021). “The Goodwin Oscillator and its Legacy”. *Acta Biotheoretica* 69.4, pp. 857–874.

Goodwin BC (1963). *Temporal organization in cells; a dynamic theory of cellular control processes.* London, Academic Press, 1963, p. 184.

Hodgkin AL & Huxley AF (1952). “A quantitative description of membrane current and its application to conduction and excitation in nerve”. *The Journal of Physiology* 117.4, pp. 500–544.

Li Z & Yang Q (2018). “Systems and synthetic biology approaches in understanding biological oscillators”. *Quantitative Biology* 6.1, pp. 1–14.

Park JH, Holló G & Schaerli Y (2024). “From resonance to chaos by modulating spatiotemporal patterns through a synthetic optogenetic oscillator”. *Nature Communications* 15.1, p. 7284.

Romano E, Baumschlager A, Akmeric EB, Palanisamy N, Houmani M, Schmidt G, Öztürk MA, Ernst L, Khammash M & Di Ventura B (2021). “Engineering AraC to make it responsive to light instead of arabinose”. *Nature chemical biology* 17.7, pp. 817–827.

Santos-Moreno J, Tasiudi E, Kusumawardhani H, Stelling J & Schaerli Y (2023). “Robustness and innovation in synthetic genotype networks”. *Nature Communications* 14.1, p. 2454.

Santos-Moreno J, Tasiudi E, Stelling J & Schaerli Y (2020). “Multistable and dynamic CRISPRi-based synthetic circuits”. *Nature Communications* 11.1, p. 2746.

Stricker J, Cookson S, Bennett MR, Mather WH, Tsimring LS & Hasty J (2008). “A fast, robust and tunable synthetic gene oscillator”. *Nature* 456.7221, pp. 516–519.

Tomazou M, Barahona M, Polizzi KM & Stan G.-B (2018). “Computational Re-design of Synthetic Genetic Oscillators for Independent Amplitude and Frequency Modulation”. *Cell Systems* 6.4, 508–520.e5.

Wilkinson DJ (2012). *Stochastic modelling for systems biology.* English. 2nd ed. Chapman and Hall/CRC mathematical & computational biology series. Boca Raton: CRC Press-Taylor & Francis.
