# Supplementary material for: A tool for modeling gene regulatory networks (GRN_modeler) and its applications to synthetic biology
Source: Mol Syst Biol. 2025 Sep 29;21(11):1618–37. doi: 10.1038/s44320-025-00148-8 (PMC12583811; doi:10.1038/s44320-025-00148-8)
Supplement: Supplementary file 2 — HTML model files [file 44320_2025_148_MOESM2_ESM.zip › SI/CFFL.html]

GRN


# Model: GRN

## Quantities

|  | Quantity Name | Type | Scope | Value | Initial Value | Units | Notes |
| --- | --- | --- | --- | --- | --- | --- | --- |
| 1 | Ecoli | compartment | GRN | 0.7 | 0.7 | micrometer^3 |  |
| 2 | mRNA\_N1 | species | Ecoli | 0 | 0 | molecule | Individual |
| 3 | P\_N1 | species | Ecoli | 0 | 0 | molecule | Individual |
| 4 | mRNA\_N2 | species | Ecoli | 0 | 0 | molecule | Individual |
| 5 | P\_N2 | species | Ecoli | 0 | 0 | molecule | Individual |
| 6 | mRNA\_N3 | species | Ecoli | 0 | 0 | molecule | Individual |
| 7 | P\_N3 | species | Ecoli | 0 | 0 | molecule | Individual |
| 8 | R1 | species | Ecoli | 30 | 30 | micromolarity | Individual |
| 9 | k0\_N1 | parameter | GRN | 0.03 | 0.03 | molecule/minute | Individual |
| 10 | k1\_N1 | parameter | GRN | 30 | 30 | molecule/minute | Individual |
| 11 | k2\_N1 | parameter | GRN | 0.34657 | 0.34657 | 1/minute | Individual |
| 12 | k3\_N1 | parameter | GRN | 6.9315 | 6.9315 | 1/minute | Individual |
| 13 | k4\_N1 | parameter | GRN | 0.069315 | 0.069315 | 1/minute | Individual |
| 14 | k0\_N2 | parameter | GRN | 0.03 | 0.03 | molecule/minute | Individual |
| 15 | k1\_N2 | parameter | GRN | 30 | 30 | molecule/minute | Individual |
| 16 | k2\_N2 | parameter | GRN | 0.34657 | 0.34657 | 1/minute | Individual |
| 17 | k3\_N2 | parameter | GRN | 6.9315 | 6.9315 | 1/minute | Individual |
| 18 | k4\_N2 | parameter | GRN | 0.069315 | 0.069315 | 1/minute | Individual |
| 19 | k0\_N3 | parameter | GRN | 0.03 | 0.03 | molecule/minute | Individual |
| 20 | k1\_N3 | parameter | GRN | 30 | 30 | molecule/minute | Individual |
| 21 | k2\_N3 | parameter | GRN | 0.34657 | 0.34657 | 1/minute | Individual |
| 22 | k3\_N3 | parameter | GRN | 6.9315 | 6.9315 | 1/minute | Individual |
| 23 | k4\_N3 | parameter | GRN | 0.069315 | 0.069315 | 1/minute | Individual |
| 24 | HILL\_N2<-N1 | parameter | GRN | 1 | 0 | dimensionless | Individual |
| 25 | K\_N2<-N1 | parameter | GRN | 40 | 40 | molecule | Individual |
| 26 | n\_N2<-N1 | parameter | GRN | 2 | 2 | dimensionless | Individual |
| 27 | HILL\_N3<-N2 | parameter | GRN | 1 | 0 | dimensionless | Individual |
| 28 | K\_N3<-N2 | parameter | GRN | 40 | 40 | molecule | Individual |
| 29 | n\_N3<-N2 | parameter | GRN | 2 | 2 | dimensionless | Individual |
| 30 | HILL\_N3<-N1 | parameter | GRN | 1 | 0 | dimensionless | Individual |
| 31 | K\_N3<-N1 | parameter | GRN | 40 | 40 | molecule | Individual |
| 32 | n\_N3<-N1 | parameter | GRN | 2 | 2 | dimensionless | Individual |
| 33 | HILL\_N1<-R1 | parameter | GRN | 1 | 0.36 | dimensionless | Individual |
| 34 | K\_out\_N1<-R1 | parameter | GRN | 40 | 40 | micromolarity | Individual |
| 35 | n\_out\_N1<-R1 | parameter | GRN | 2 | 2 | dimensionless | Individual |

## Repeated Assignments

|  | Repeated Assignments | Initial Value | Notes |
| --- | --- | --- | --- |
| 1 | [HILL\_N2<-N1] = (P\_N1/[K\_N2<-N1])^[n\_N2<-N1]/(1+(P\_N1/[K\_N2<-N1])^[n\_N2<-N1]) | 0 | Individual |
| 2 | [HILL\_N3<-N2] = (P\_N2/[K\_N3<-N2])^[n\_N3<-N2]/(1+(P\_N2/[K\_N3<-N2])^[n\_N3<-N2]) | 0 | Individual |
| 3 | [HILL\_N3<-N1] = (P\_N1/[K\_N3<-N1])^[n\_N3<-N1]/(1+(P\_N1/[K\_N3<-N1])^[n\_N3<-N1]) | 0 | Individual |
| 4 | [HILL\_N1<-R1] = (R1/[K\_out\_N1<-R1])^[n\_out\_N1<-R1]/(1+(R1/[K\_out\_N1<-R1])^[n\_out\_N1<-R1]) | 0.36 | Individual |

## Reactions

|  | Reactions | Notes |
| --- | --- | --- |
| 1 | null <-> mRNA\_N1 | Individual |
|  | k0\_N1+k1\_N1\*[HILL\_N1<-R1]-k2\_N1\*mRNA\_N1 |  |
| 2 | null <-> P\_N1 | Individual |
|  | k3\_N1\*mRNA\_N1-k4\_N1\*P\_N1 |  |
| 3 | null <-> mRNA\_N2 | Individual |
|  | k0\_N2+k1\_N2\*[HILL\_N2<-N1]-k2\_N2\*mRNA\_N2 |  |
| 4 | null <-> P\_N2 | Individual |
|  | k3\_N2\*mRNA\_N2-k4\_N2\*P\_N2 |  |
| 5 | null <-> mRNA\_N3 | Individual |
|  | k0\_N3+k1\_N3\*[HILL\_N3<-N2]\*[HILL\_N3<-N1]-k2\_N3\*mRNA\_N3 |  |
| 6 | null <-> P\_N3 | Individual |
|  | k3\_N3\*mRNA\_N3-k4\_N3\*P\_N3 |  |

# Model Equations

## ODEs

|  | ODEs |
| --- | --- |
| 1 | d(mRNA\_N1)/dt = (k0\_N1+k1\_N1\*[HILL\_N1<-R1]-k2\_N1\*mRNA\_N1) |
| 2 | d(P\_N1)/dt = (k3\_N1\*mRNA\_N1-k4\_N1\*P\_N1) |
| 3 | d(mRNA\_N2)/dt = (k0\_N2+k1\_N2\*[HILL\_N2<-N1]-k2\_N2\*mRNA\_N2) |
| 4 | d(P\_N2)/dt = (k3\_N2\*mRNA\_N2-k4\_N2\*P\_N2) |
| 5 | d(mRNA\_N3)/dt = (k0\_N3+k1\_N3\*[HILL\_N3<-N2]\*[HILL\_N3<-N1]-k2\_N3\*mRNA\_N3) |
| 6 | d(P\_N3)/dt = (k3\_N3\*mRNA\_N3-k4\_N3\*P\_N3) |

Report generated by SimBiology v. 23.2 (R2023b) on 08-Aug-2024 14:45:45
