# Supplementary material for: A tool for modeling gene regulatory networks (GRN_modeler) and its applications to synthetic biology
Source: Mol Syst Biol. 2025 Sep 29;21(11):1618–37. doi: 10.1038/s44320-025-00148-8 (PMC12583811; doi:10.1038/s44320-025-00148-8)
Supplement: Supplementary file 2 — HTML model files [file 44320_2025_148_MOESM2_ESM.zip › SI/goodwin.html]

GRN


# Model: GRN

## Quantities

|  | Quantity Name | Type | Scope | Value | Initial Value | Units | Notes |
| --- | --- | --- | --- | --- | --- | --- | --- |
| 1 | Ecoli | compartment | GRN | 0.7 | 0.7 | micrometer^3 |  |
| 2 | mRNA\_N1 | species | Ecoli | 0 | 0 | molecule | Individual |
| 3 | uP\_N1 | species | Ecoli | 0 | 0 | molecule | Individual |
| 4 | P\_N1 | species | Ecoli | 0 | 0 | molecule | Individual |
| 5 | PROT1 | species | Ecoli | 50 | 50 | molecule | Individual |
| 6 | n\_copy\_N1 | parameter | GRN | 25 | 25 | molecule | Individual |
| 7 | a0\_N1 | parameter | GRN | 0.001 | 0.001 | 1/minute | Individual |
| 8 | a1\_N1 | parameter | GRN | 100 | 100 | 1/minute | Individual |
| 9 | k\_mRNA\_degr | parameter | GRN | 0.5 | 0.5 | 1/minute | Common |
| 10 | k\_translation\_N1 | parameter | GRN | 50 | 50 | 1/minute | Individual |
| 11 | k\_mat | parameter | GRN | 0.4 | 0.4 | 1/minute | Common |
| 12 | dilution | parameter | GRN | 0.01 | 0.01 | 1/minute | Common |
| 13 | HILL\_N1|-N1 | parameter | GRN | 1 | 1 | dimensionless | Individual |
| 14 | K\_molecule\_N1|-N1 | parameter | GRN | 5 | 5 | molecule | Individual |
| 15 | n\_molecule\_N1|-N1 | parameter | GRN | 2 | 2 | dimensionless | Individual |
| 16 | K\_protease | parameter | GRN | 30 | 30 | molecule | Common |
| 17 | k\_protease\_max | parameter | GRN | 50 | 50 | 1/minute | Common |
| 18 | protease\_rate\_PROT1 | parameter | GRN | 0 | 83.3333 | 1/minute | Individual |
| 19 | Substrates\_PROT1 | parameter | GRN | 0 | 0 | molecule | Individual |

## Repeated Assignments

|  | Repeated Assignments | Initial Value | Notes |
| --- | --- | --- | --- |
| 1 | [HILL\_N1|-N1] = 1/(1+(P\_N1/[K\_molecule\_N1|-N1])^[n\_molecule\_N1|-N1]) | 1 | Individual |
| 2 | protease\_rate\_PROT1 = k\_protease\_max\*PROT1/(K\_protease+Substrates\_PROT1) | 83.3333 | Individual |
| 3 | Substrates\_PROT1 = uP\_N1+P\_N1 | 0 | Individual |

## Reactions

|  | Reactions | Notes |
| --- | --- | --- |
| 1 | null <-> mRNA\_N1 | Individual |
|  | n\_copy\_N1\*(a0\_N1+a1\_N1\*[HILL\_N1|-N1])-(k\_mRNA\_degr+dilution)\*mRNA\_N1 |  |
| 2 | null <-> uP\_N1 | Individual |
|  | k\_translation\_N1\*mRNA\_N1-(dilution)\*uP\_N1 |  |
| 3 | uP\_N1 -> P\_N1 | Individual |
|  | k\_mat\*uP\_N1 |  |
| 4 | null <-> P\_N1 | Individual |
|  | -(dilution)\*P\_N1 |  |
| 5 | uP\_N1 -> null | Individual |
|  | protease\_rate\_PROT1\*uP\_N1 |  |
| 6 | P\_N1 -> null | Individual |
|  | protease\_rate\_PROT1\*P\_N1 |  |

# Model Equations

## ODEs

|  | ODEs |
| --- | --- |
| 1 | d(mRNA\_N1)/dt = (n\_copy\_N1\*(a0\_N1+a1\_N1\*[HILL\_N1|-N1])-(k\_mRNA\_degr+dilution)\*mRNA\_N1) |
| 2 | d(uP\_N1)/dt = (k\_translation\_N1\*mRNA\_N1-(dilution)\*uP\_N1) - (k\_mat\*uP\_N1) - (protease\_rate\_PROT1\*uP\_N1) |
| 3 | d(P\_N1)/dt = (k\_mat\*uP\_N1) + (-(dilution)\*P\_N1) - (protease\_rate\_PROT1\*P\_N1) |

Report generated by SimBiology v. 23.2 (R2023b) on 24-Mar-2025 10:35:46
