# Supplementary material for: A tool for modeling gene regulatory networks (GRN_modeler) and its applications to synthetic biology
Source: Mol Syst Biol. 2025 Sep 29;21(11):1618–37. doi: 10.1038/s44320-025-00148-8 (PMC12583811; doi:10.1038/s44320-025-00148-8)
Supplement: Supplementary file 2 — HTML model files [file 44320_2025_148_MOESM2_ESM.zip › SI/light_sensor.html]

GRN


# Model: GRN

## Quantities

|  | Quantity Name | Type | Scope | Value | Initial Value | Units | Notes |
| --- | --- | --- | --- | --- | --- | --- | --- |
| 1 | Ecoli | compartment | GRN | 0.7 | 0.7 | micrometer^3 |  |
| 2 | dCas | species | Ecoli | 1434 | 1434 | molecule | Common |
| 3 | sgRNA\_N1 | species | Ecoli | 10 | 10 | molecule | Individual |
| 4 | dCas:sgRNA\_N1 | species | Ecoli | 0 | 0 | molecule | Individual |
| 5 | DNA\_N1 | species | Ecoli | 30 | 30 | molecule | Individual |
| 6 | mRNA\_N1 | species | Ecoli | 0 | 0 | molecule | Individual |
| 7 | P\_N1 | species | Ecoli | 0 | 0 | molecule | Individual |
| 8 | Light | species | Ecoli | 0 | 50 | micromolarity | Individual |
| 9 | Ara | species | Ecoli | 0 | 0 | micromolarity | Individual |
| 10 | sgRNA\_N2 | species | Ecoli | 0 | 0 | molecule | Individual |
| 11 | dCas:sgRNA\_N2 | species | Ecoli | 0 | 0 | molecule | Individual |
| 12 | DNA\_N2 | species | Ecoli | 30 | 30 | molecule | Individual |
| 13 | mRNA\_N2 | species | Ecoli | 0 | 0 | molecule | Individual |
| 14 | P\_N2 | species | Ecoli | 0 | 0 | molecule | Individual |
| 15 | dCas:sgRNA\_N1:DNA\_N2 | species | Ecoli | 0 | 0 | molecule | Individual |
| 16 | sgRNA\_N3 | species | Ecoli | 0 | 0 | molecule | Individual |
| 17 | dCas:sgRNA\_N3 | species | Ecoli | 0 | 0 | molecule | Individual |
| 18 | DNA\_N3 | species | Ecoli | 30 | 30 | molecule | Individual |
| 19 | mRNA\_N3 | species | Ecoli | 0 | 0 | molecule | Individual |
| 20 | P\_N3 | species | Ecoli | 0 | 0 | molecule | Individual |
| 21 | dCas:sgRNA\_N2:DNA\_N3 | species | Ecoli | 0 | 0 | molecule | Individual |
| 22 | dCas:sgRNA\_N3:DNA\_N1 | species | Ecoli | 0 | 0 | molecule | Individual |
| 23 | dilution | parameter | GRN | 0.005 | 0.005 | 1/minute | Common |
| 24 | k\_P | parameter | GRN | 6.9315 | 6.9315 | 1/minute | Common |
| 25 | d\_P | parameter | GRN | 0.051315 | 0.051315 | 1/minute | Common |
| 26 | a0 | parameter | GRN | 0.03 | 0.03 | molecule/minute | Common |
| 27 | a1\_N1 | parameter | GRN | 2.36 | 2.36 | 1/minute | Individual |
| 28 | d\_RNA | parameter | GRN | 0.32857 | 0.32857 | 1/minute | Common |
| 29 | kfds | parameter | GRN | 1.4674 | 1.4674 | 1/(molecule\*minute) | Common |
| 30 | krds | parameter | GRN | 0.077625 | 0.077625 | 1/minute | Common |
| 31 | leak | parameter | GRN | 0.01 | 0.01 | dimensionless | Common |
| 32 | HILL\_N1HILL<-R1 | parameter | GRN | 1 | 0.077682 | dimensionless | Individual |
| 33 | k\_light\_ratio\_N1HILL<-R1 | parameter | GRN | 0.31854 | 0.31854 | dimensionless | Individual |
| 34 | K\_light\_N1HILL<-R1 | parameter | GRN | 156.8606 | 156.8606 | micromolarity | Individual |
| 35 | n\_light\_N1HILL<-R1 | parameter | GRN | 0.98973 | 0.98973 | dimensionless | Individual |
| 36 | K\_ara\_N1HILL<-R1 | parameter | GRN | 0.00027567 | 0.00027567 | micromolarity | Individual |
| 37 | n\_ara\_N1HILL<-R1 | parameter | GRN | 1.1177 | 1.1177 | dimensionless | Individual |
| 38 | HILL\_light\_N1HILL<-R1HILL\_light<-Light | parameter | GRN | 1 | 50 | micromolarity | Individual |
| 39 | HILL\_ara\_N1HILL<-R1HILL\_ara<-Ara | parameter | GRN | 1 | 0 | micromolarity | Individual |
| 40 | A | parameter | GRN | 100 | 100 | micromolarity | Individual |
| 41 | T | parameter | GRN | 1440 | 1440 | minute | Individual |
| 42 | a1\_N2 | parameter | GRN | 1 | 1 | 1/minute | Individual |
| 43 | kfdsd | parameter | GRN | 0.26702 | 0.26702 | 1/(molecule\*minute) | Common |
| 44 | krdsd | parameter | GRN | 0.7762 | 0.7762 | 1/minute | Common |
| 45 | a1\_N3 | parameter | GRN | 1 | 1 | 1/minute | Individual |

## Repeated Assignments

|  | Repeated Assignments | Initial Value | Notes |
| --- | --- | --- | --- |
| 1 | [HILL\_N1HILL<-R1] = (([HILL\_ara\_N1HILL<-R1HILL\_ara<-Ara]/[K\_ara\_N1HILL<-R1])^[n\_ara\_N1HILL<-R1]+[k\_light\_ratio\_N1HILL<-R1]\*([HILL\_light\_N1HILL<-R1HILL\_light<-Light]/[K\_light\_N1HILL<-R1])^[n\_light\_N1HILL<-R1])/((1+([HILL\_ara\_N1HILL<-R1HILL\_ara<-Ara]/[K\_ara\_N1HILL<-R1])^[n\_ara\_N1HILL<-R1])\*(1+([HILL\_light\_N1HILL<-R1HILL\_light<-Light]/[K\_light\_N1HILL<-R1])^[n\_light\_N1HILL<-R1])) | 0.077682 | Individual |
| 2 | [HILL\_light\_N1HILL<-R1HILL\_light<-Light] = Light | 50 | Individual |
| 3 | [HILL\_ara\_N1HILL<-R1HILL\_ara<-Ara] = Ara | 0 | Individual |
| 4 | Light = A\*(1+sign(sin(2\*pi/T\*time)))/2 | 50 |  |

## Reactions

|  | Reactions | Notes |
| --- | --- | --- |
| 1 | null <-> mRNA\_N1 | Individual |
|  | a0+a1\_N1\*(leak+(1-leak)\*[HILL\_N1HILL<-R1])\*DNA\_N1-(dilution+d\_RNA)\*mRNA\_N1 |  |
| 2 | null <-> P\_N1 | Individual |
|  | k\_P\*mRNA\_N1-(dilution+d\_P)\*P\_N1 |  |
| 3 | null <-> sgRNA\_N1 | Individual |
|  | a0+a1\_N1\*(leak+(1-leak)\*[HILL\_N1HILL<-R1])\*DNA\_N1-(dilution+d\_RNA)\*sgRNA\_N1 |  |
| 4 | dCas + sgRNA\_N1 <-> [dCas:sgRNA\_N1] | Individual |
|  | kfds\*dCas\*sgRNA\_N1-krds\*[dCas:sgRNA\_N1] |  |
| 5 | [dCas:sgRNA\_N1] -> dCas | Individual |
|  | dilution\*[dCas:sgRNA\_N1] |  |
| 6 | null <-> mRNA\_N2 | Individual |
|  | a0+a1\_N2\*(leak+(1-leak))\*DNA\_N2-(dilution+d\_RNA)\*mRNA\_N2 |  |
| 7 | null <-> P\_N2 | Individual |
|  | k\_P\*mRNA\_N2-(dilution+d\_P)\*P\_N2 |  |
| 8 | null <-> sgRNA\_N2 | Individual |
|  | a0+a1\_N2\*(leak+(1-leak))\*DNA\_N2-(dilution+d\_RNA)\*sgRNA\_N2 |  |
| 9 | dCas + sgRNA\_N2 <-> [dCas:sgRNA\_N2] | Individual |
|  | kfds\*dCas\*sgRNA\_N2-krds\*[dCas:sgRNA\_N2] |  |
| 10 | [dCas:sgRNA\_N2] -> dCas | Individual |
|  | dilution\*[dCas:sgRNA\_N2] |  |
| 11 | [dCas:sgRNA\_N1] + DNA\_N2 <-> [dCas:sgRNA\_N1:DNA\_N2] | Individual |
|  | kfdsd\*[dCas:sgRNA\_N1]\*DNA\_N2-krdsd\*[dCas:sgRNA\_N1:DNA\_N2] |  |
| 12 | [dCas:sgRNA\_N1:DNA\_N2] -> dCas + DNA\_N2 | Individual |
|  | dilution\*[dCas:sgRNA\_N1:DNA\_N2] |  |
| 13 | null <-> mRNA\_N3 | Individual |
|  | a0+a1\_N3\*(leak+(1-leak))\*DNA\_N3-(dilution+d\_RNA)\*mRNA\_N3 |  |
| 14 | null <-> P\_N3 | Individual |
|  | k\_P\*mRNA\_N3-(dilution+d\_P)\*P\_N3 |  |
| 15 | null <-> sgRNA\_N3 | Individual |
|  | a0+a1\_N3\*(leak+(1-leak))\*DNA\_N3-(dilution+d\_RNA)\*sgRNA\_N3 |  |
| 16 | dCas + sgRNA\_N3 <-> [dCas:sgRNA\_N3] | Individual |
|  | kfds\*dCas\*sgRNA\_N3-krds\*[dCas:sgRNA\_N3] |  |
| 17 | [dCas:sgRNA\_N3] -> dCas | Individual |
|  | dilution\*[dCas:sgRNA\_N3] |  |
| 18 | [dCas:sgRNA\_N2] + DNA\_N3 <-> [dCas:sgRNA\_N2:DNA\_N3] | Individual |
|  | kfdsd\*[dCas:sgRNA\_N2]\*DNA\_N3-krdsd\*[dCas:sgRNA\_N2:DNA\_N3] |  |
| 19 | [dCas:sgRNA\_N2:DNA\_N3] -> dCas + DNA\_N3 | Individual |
|  | dilution\*[dCas:sgRNA\_N2:DNA\_N3] |  |
| 20 | [dCas:sgRNA\_N3] + DNA\_N1 <-> [dCas:sgRNA\_N3:DNA\_N1] | Individual |
|  | kfdsd\*[dCas:sgRNA\_N3]\*DNA\_N1-krdsd\*[dCas:sgRNA\_N3:DNA\_N1] |  |
| 21 | [dCas:sgRNA\_N3:DNA\_N1] -> dCas + DNA\_N1 | Individual |
|  | dilution\*[dCas:sgRNA\_N3:DNA\_N1] |  |

# Model Equations

## ODEs

|  | ODEs |
| --- | --- |
| 1 | d(dCas)/dt = -(kfds\*dCas\*sgRNA\_N1-krds\*[dCas:sgRNA\_N1]) + (dilution\*[dCas:sgRNA\_N1]) - (kfds\*dCas\*sgRNA\_N2-krds\*[dCas:sgRNA\_N2]) + (dilution\*[dCas:sgRNA\_N2]) + (dilution\*[dCas:sgRNA\_N1:DNA\_N2]) - (kfds\*dCas\*sgRNA\_N3-krds\*[dCas:sgRNA\_N3]) + (dilution\*[dCas:sgRNA\_N3]) + (dilution\*[dCas:sgRNA\_N2:DNA\_N3]) + (dilution\*[dCas:sgRNA\_N3:DNA\_N1]) |
| 2 | d(sgRNA\_N1)/dt = (a0+a1\_N1\*(leak+(1-leak)\*[HILL\_N1HILL<-R1])\*DNA\_N1-(dilution+d\_RNA)\*sgRNA\_N1) - (kfds\*dCas\*sgRNA\_N1-krds\*[dCas:sgRNA\_N1]) |
| 3 | d([dCas:sgRNA\_N1])/dt = (kfds\*dCas\*sgRNA\_N1-krds\*[dCas:sgRNA\_N1]) - (dilution\*[dCas:sgRNA\_N1]) - (kfdsd\*[dCas:sgRNA\_N1]\*DNA\_N2-krdsd\*[dCas:sgRNA\_N1:DNA\_N2]) |
| 4 | d(DNA\_N1)/dt = -(kfdsd\*[dCas:sgRNA\_N3]\*DNA\_N1-krdsd\*[dCas:sgRNA\_N3:DNA\_N1]) + (dilution\*[dCas:sgRNA\_N3:DNA\_N1]) |
| 5 | d(mRNA\_N1)/dt = (a0+a1\_N1\*(leak+(1-leak)\*[HILL\_N1HILL<-R1])\*DNA\_N1-(dilution+d\_RNA)\*mRNA\_N1) |
| 6 | d(P\_N1)/dt = (k\_P\*mRNA\_N1-(dilution+d\_P)\*P\_N1) |
| 7 | d(sgRNA\_N2)/dt = (a0+a1\_N2\*(leak+(1-leak))\*DNA\_N2-(dilution+d\_RNA)\*sgRNA\_N2) - (kfds\*dCas\*sgRNA\_N2-krds\*[dCas:sgRNA\_N2]) |
| 8 | d([dCas:sgRNA\_N2])/dt = (kfds\*dCas\*sgRNA\_N2-krds\*[dCas:sgRNA\_N2]) - (dilution\*[dCas:sgRNA\_N2]) - (kfdsd\*[dCas:sgRNA\_N2]\*DNA\_N3-krdsd\*[dCas:sgRNA\_N2:DNA\_N3]) |
| 9 | d(DNA\_N2)/dt = -(kfdsd\*[dCas:sgRNA\_N1]\*DNA\_N2-krdsd\*[dCas:sgRNA\_N1:DNA\_N2]) + (dilution\*[dCas:sgRNA\_N1:DNA\_N2]) |
| 10 | d(mRNA\_N2)/dt = (a0+a1\_N2\*(leak+(1-leak))\*DNA\_N2-(dilution+d\_RNA)\*mRNA\_N2) |
| 11 | d(P\_N2)/dt = (k\_P\*mRNA\_N2-(dilution+d\_P)\*P\_N2) |
| 12 | d([dCas:sgRNA\_N1:DNA\_N2])/dt = (kfdsd\*[dCas:sgRNA\_N1]\*DNA\_N2-krdsd\*[dCas:sgRNA\_N1:DNA\_N2]) - (dilution\*[dCas:sgRNA\_N1:DNA\_N2]) |
| 13 | d(sgRNA\_N3)/dt = (a0+a1\_N3\*(leak+(1-leak))\*DNA\_N3-(dilution+d\_RNA)\*sgRNA\_N3) - (kfds\*dCas\*sgRNA\_N3-krds\*[dCas:sgRNA\_N3]) |
| 14 | d([dCas:sgRNA\_N3])/dt = (kfds\*dCas\*sgRNA\_N3-krds\*[dCas:sgRNA\_N3]) - (dilution\*[dCas:sgRNA\_N3]) - (kfdsd\*[dCas:sgRNA\_N3]\*DNA\_N1-krdsd\*[dCas:sgRNA\_N3:DNA\_N1]) |
| 15 | d(DNA\_N3)/dt = -(kfdsd\*[dCas:sgRNA\_N2]\*DNA\_N3-krdsd\*[dCas:sgRNA\_N2:DNA\_N3]) + (dilution\*[dCas:sgRNA\_N2:DNA\_N3]) |
| 16 | d(mRNA\_N3)/dt = (a0+a1\_N3\*(leak+(1-leak))\*DNA\_N3-(dilution+d\_RNA)\*mRNA\_N3) |
| 17 | d(P\_N3)/dt = (k\_P\*mRNA\_N3-(dilution+d\_P)\*P\_N3) |
| 18 | d([dCas:sgRNA\_N2:DNA\_N3])/dt = (kfdsd\*[dCas:sgRNA\_N2]\*DNA\_N3-krdsd\*[dCas:sgRNA\_N2:DNA\_N3]) - (dilution\*[dCas:sgRNA\_N2:DNA\_N3]) |
| 19 | d([dCas:sgRNA\_N3:DNA\_N1])/dt = (kfdsd\*[dCas:sgRNA\_N3]\*DNA\_N1-krdsd\*[dCas:sgRNA\_N3:DNA\_N1]) - (dilution\*[dCas:sgRNA\_N3:DNA\_N1]) |

Report generated by SimBiology v. 23.2 (R2023b) on 22-Oct-2024 08:56:46
