# Supplementary material for: A tool for modeling gene regulatory networks (GRN_modeler) and its applications to synthetic biology
Source: Mol Syst Biol. 2025 Sep 29;21(11):1618–37. doi: 10.1038/s44320-025-00148-8 (PMC12583811; doi:10.1038/s44320-025-00148-8)
Supplement: Supplementary file 2 — HTML model files [file 44320_2025_148_MOESM2_ESM.zip › SI/N6.html]

GRN


# Model: GRN

## Quantities

|  | Quantity Name | Type | Scope | Value | Initial Value | Units | Notes |
| --- | --- | --- | --- | --- | --- | --- | --- |
| 1 | Ecoli | compartment | GRN | 0.7 | 0.7 | micrometer^3 |  |
| 2 | mRNA\_N1 | species | Ecoli | 0 | 0 | molecule | Individual |
| 3 | P\_N1 | species | Ecoli | 1000 | 1000 | molecule | Individual |
| 4 | mRNA\_N2 | species | Ecoli | 0 | 0 | molecule | Individual |
| 5 | P\_N2 | species | Ecoli | 0 | 0 | molecule | Individual |
| 6 | mRNA\_N3 | species | Ecoli | 0 | 0 | molecule | Individual |
| 7 | P\_N3 | species | Ecoli | 0 | 0 | molecule | Individual |
| 8 | mRNA\_N4 | species | Ecoli | 0 | 0 | molecule | Individual |
| 9 | P\_N4 | species | Ecoli | 0 | 0 | molecule | Individual |
| 10 | mRNA\_N5 | species | Ecoli | 0 | 0 | molecule | Individual |
| 11 | P\_N5 | species | Ecoli | 0 | 0 | molecule | Individual |
| 12 | mRNA\_N6 | species | Ecoli | 0 | 0 | molecule | Individual |
| 13 | P\_N6 | species | Ecoli | 0 | 0 | molecule | Individual |
| 14 | k0\_N1 | parameter | GRN | 0.03 | 0.03 | molecule/minute | Individual |
| 15 | k1\_N1 | parameter | GRN | 30 | 30 | molecule/minute | Individual |
| 16 | k2\_N1 | parameter | GRN | 0.34657 | 0.34657 | 1/minute | Individual |
| 17 | k3\_N1 | parameter | GRN | 6.9315 | 6.9315 | 1/minute | Individual |
| 18 | k4\_N1 | parameter | GRN | 0.069315 | 0.069315 | 1/minute | Individual |
| 19 | k0\_N2 | parameter | GRN | 0.03 | 0.03 | molecule/minute | Individual |
| 20 | k1\_N2 | parameter | GRN | 30 | 30 | molecule/minute | Individual |
| 21 | k2\_N2 | parameter | GRN | 0.34657 | 0.34657 | 1/minute | Individual |
| 22 | k3\_N2 | parameter | GRN | 6.9315 | 6.9315 | 1/minute | Individual |
| 23 | k4\_N2 | parameter | GRN | 0.069315 | 0.069315 | 1/minute | Individual |
| 24 | k0\_N3 | parameter | GRN | 0.03 | 0.03 | molecule/minute | Individual |
| 25 | k1\_N3 | parameter | GRN | 30 | 30 | molecule/minute | Individual |
| 26 | k2\_N3 | parameter | GRN | 0.34657 | 0.34657 | 1/minute | Individual |
| 27 | k3\_N3 | parameter | GRN | 6.9315 | 6.9315 | 1/minute | Individual |
| 28 | k4\_N3 | parameter | GRN | 0.069315 | 0.069315 | 1/minute | Individual |
| 29 | k0\_N4 | parameter | GRN | 0.03 | 0.03 | molecule/minute | Individual |
| 30 | k1\_N4 | parameter | GRN | 30 | 30 | molecule/minute | Individual |
| 31 | k2\_N4 | parameter | GRN | 0.34657 | 0.34657 | 1/minute | Individual |
| 32 | k3\_N4 | parameter | GRN | 6.9315 | 6.9315 | 1/minute | Individual |
| 33 | k4\_N4 | parameter | GRN | 0.069315 | 0.069315 | 1/minute | Individual |
| 34 | k0\_N5 | parameter | GRN | 0.03 | 0.03 | molecule/minute | Individual |
| 35 | k1\_N5 | parameter | GRN | 30 | 30 | molecule/minute | Individual |
| 36 | k2\_N5 | parameter | GRN | 0.34657 | 0.34657 | 1/minute | Individual |
| 37 | k3\_N5 | parameter | GRN | 6.9315 | 6.9315 | 1/minute | Individual |
| 38 | k4\_N5 | parameter | GRN | 0.069315 | 0.069315 | 1/minute | Individual |
| 39 | k0\_N6 | parameter | GRN | 0.03 | 0.03 | molecule/minute | Individual |
| 40 | k1\_N6 | parameter | GRN | 30 | 30 | molecule/minute | Individual |
| 41 | k2\_N6 | parameter | GRN | 0.34657 | 0.34657 | 1/minute | Individual |
| 42 | k3\_N6 | parameter | GRN | 6.9315 | 6.9315 | 1/minute | Individual |
| 43 | k4\_N6 | parameter | GRN | 0.069315 | 0.069315 | 1/minute | Individual |
| 44 | HILL\_N2|-N1 | parameter | GRN | 1 | 0.0015974 | dimensionless | Individual |
| 45 | K\_N2|-N1 | parameter | GRN | 40 | 40 | molecule | Individual |
| 46 | n\_N2|-N1 | parameter | GRN | 2 | 2 | dimensionless | Individual |
| 47 | HILL\_N3|-N2 | parameter | GRN | 1 | 1 | dimensionless | Individual |
| 48 | K\_N3|-N2 | parameter | GRN | 40 | 40 | molecule | Individual |
| 49 | n\_N3|-N2 | parameter | GRN | 2 | 2 | dimensionless | Individual |
| 50 | HILL\_N4|-N3 | parameter | GRN | 1 | 1 | dimensionless | Individual |
| 51 | K\_N4|-N3 | parameter | GRN | 40 | 40 | molecule | Individual |
| 52 | n\_N4|-N3 | parameter | GRN | 2 | 2 | dimensionless | Individual |
| 53 | HILL\_N5|-N4 | parameter | GRN | 1 | 1 | dimensionless | Individual |
| 54 | K\_N5|-N4 | parameter | GRN | 40 | 40 | molecule | Individual |
| 55 | n\_N5|-N4 | parameter | GRN | 2 | 2 | dimensionless | Individual |
| 56 | HILL\_N6|-N5 | parameter | GRN | 1 | 1 | dimensionless | Individual |
| 57 | K\_N6|-N5 | parameter | GRN | 40 | 40 | molecule | Individual |
| 58 | n\_N6|-N5 | parameter | GRN | 2 | 2 | dimensionless | Individual |
| 59 | HILL\_N1|-N6 | parameter | GRN | 1 | 1 | dimensionless | Individual |
| 60 | K\_N1|-N6 | parameter | GRN | 40 | 40 | molecule | Individual |
| 61 | n\_N1|-N6 | parameter | GRN | 2 | 2 | dimensionless | Individual |
| 62 | HILL\_N4|-N1 | parameter | GRN | 1 | 0.0015974 | dimensionless | Individual |
| 63 | K\_N4|-N1 | parameter | GRN | 40 | 40 | molecule | Individual |
| 64 | n\_N4|-N1 | parameter | GRN | 2 | 2 | dimensionless | Individual |
| 65 | HILL\_N1|-N4 | parameter | GRN | 1 | 1 | dimensionless | Individual |
| 66 | K\_N1|-N4 | parameter | GRN | 40 | 40 | molecule | Individual |
| 67 | n\_N1|-N4 | parameter | GRN | 2 | 2 | dimensionless | Individual |
| 68 | HILL\_N5|-N2 | parameter | GRN | 1 | 1 | dimensionless | Individual |
| 69 | K\_N5|-N2 | parameter | GRN | 40 | 40 | molecule | Individual |
| 70 | n\_N5|-N2 | parameter | GRN | 2 | 2 | dimensionless | Individual |
| 71 | HILL\_N2|-N5 | parameter | GRN | 1 | 1 | dimensionless | Individual |
| 72 | K\_N2|-N5 | parameter | GRN | 40 | 40 | molecule | Individual |
| 73 | n\_N2|-N5 | parameter | GRN | 2 | 2 | dimensionless | Individual |
| 74 | HILL\_N6|-N3 | parameter | GRN | 1 | 1 | dimensionless | Individual |
| 75 | K\_N6|-N3 | parameter | GRN | 40 | 40 | molecule | Individual |
| 76 | n\_N6|-N3 | parameter | GRN | 2 | 2 | dimensionless | Individual |
| 77 | HILL\_N3|-N6 | parameter | GRN | 1 | 1 | dimensionless | Individual |
| 78 | K\_N3|-N6 | parameter | GRN | 40 | 40 | molecule | Individual |
| 79 | n\_N3|-N6 | parameter | GRN | 2 | 2 | dimensionless | Individual |

## Repeated Assignments

|  | Repeated Assignments | Initial Value | Notes |
| --- | --- | --- | --- |
| 1 | [HILL\_N2|-N1] = 1/(1+(P\_N1/[K\_N2|-N1])^[n\_N2|-N1]) | 0.0015974 | Individual |
| 2 | [HILL\_N3|-N2] = 1/(1+(P\_N2/[K\_N3|-N2])^[n\_N3|-N2]) | 1 | Individual |
| 3 | [HILL\_N4|-N3] = 1/(1+(P\_N3/[K\_N4|-N3])^[n\_N4|-N3]) | 1 | Individual |
| 4 | [HILL\_N5|-N4] = 1/(1+(P\_N4/[K\_N5|-N4])^[n\_N5|-N4]) | 1 | Individual |
| 5 | [HILL\_N6|-N5] = 1/(1+(P\_N5/[K\_N6|-N5])^[n\_N6|-N5]) | 1 | Individual |
| 6 | [HILL\_N1|-N6] = 1/(1+(P\_N6/[K\_N1|-N6])^[n\_N1|-N6]) | 1 | Individual |
| 7 | [HILL\_N4|-N1] = 1/(1+(P\_N1/[K\_N4|-N1])^[n\_N4|-N1]) | 0.0015974 | Individual |
| 8 | [HILL\_N1|-N4] = 1/(1+(P\_N4/[K\_N1|-N4])^[n\_N1|-N4]) | 1 | Individual |
| 9 | [HILL\_N5|-N2] = 1/(1+(P\_N2/[K\_N5|-N2])^[n\_N5|-N2]) | 1 | Individual |
| 10 | [HILL\_N2|-N5] = 1/(1+(P\_N5/[K\_N2|-N5])^[n\_N2|-N5]) | 1 | Individual |
| 11 | [HILL\_N6|-N3] = 1/(1+(P\_N3/[K\_N6|-N3])^[n\_N6|-N3]) | 1 | Individual |
| 12 | [HILL\_N3|-N6] = 1/(1+(P\_N6/[K\_N3|-N6])^[n\_N3|-N6]) | 1 | Individual |

## Reactions

|  | Reactions |
| --- | --- |
| 1 | null -> mRNA\_N1 |
|  | k0\_N1+k1\_N1\*[HILL\_N1|-N6]\*[HILL\_N1|-N4] |
| 2 | mRNA\_N1 -> null |
|  | -(-k2\_N1\*mRNA\_N1) |
| 3 | mRNA\_N1 -> P\_N1 + mRNA\_N1 |
|  | k3\_N1\*mRNA\_N1 |
| 4 | P\_N1 -> null |
|  | -(-k4\_N1\*P\_N1) |
| 5 | null -> mRNA\_N2 |
|  | k0\_N2+k1\_N2\*[HILL\_N2|-N1]\*[HILL\_N2|-N5] |
| 6 | mRNA\_N2 -> null |
|  | -(-k2\_N2\*mRNA\_N2) |
| 7 | mRNA\_N2 -> P\_N2 + mRNA\_N2 |
|  | k3\_N2\*mRNA\_N2 |
| 8 | P\_N2 -> null |
|  | -(-k4\_N2\*P\_N2) |
| 9 | null -> mRNA\_N3 |
|  | k0\_N3+k1\_N3\*[HILL\_N3|-N2]\*[HILL\_N3|-N6] |
| 10 | mRNA\_N3 -> null |
|  | -(-k2\_N3\*mRNA\_N3) |
| 11 | mRNA\_N3 -> P\_N3 + mRNA\_N3 |
|  | k3\_N3\*mRNA\_N3 |
| 12 | P\_N3 -> null |
|  | -(-k4\_N3\*P\_N3) |
| 13 | null -> mRNA\_N4 |
|  | k0\_N4+k1\_N4\*[HILL\_N4|-N3]\*[HILL\_N4|-N1] |
| 14 | mRNA\_N4 -> null |
|  | -(-k2\_N4\*mRNA\_N4) |
| 15 | mRNA\_N4 -> P\_N4 + mRNA\_N4 |
|  | k3\_N4\*mRNA\_N4 |
| 16 | P\_N4 -> null |
|  | -(-k4\_N4\*P\_N4) |
| 17 | null -> mRNA\_N5 |
|  | k0\_N5+k1\_N5\*[HILL\_N5|-N4]\*[HILL\_N5|-N2] |
| 18 | mRNA\_N5 -> null |
|  | -(-k2\_N5\*mRNA\_N5) |
| 19 | mRNA\_N5 -> P\_N5 + mRNA\_N5 |
|  | k3\_N5\*mRNA\_N5 |
| 20 | P\_N5 -> null |
|  | -(-k4\_N5\*P\_N5) |
| 21 | null -> mRNA\_N6 |
|  | k0\_N6+k1\_N6\*[HILL\_N6|-N5]\*[HILL\_N6|-N3] |
| 22 | mRNA\_N6 -> null |
|  | -(-k2\_N6\*mRNA\_N6) |
| 23 | mRNA\_N6 -> P\_N6 + mRNA\_N6 |
|  | k3\_N6\*mRNA\_N6 |
| 24 | P\_N6 -> null |
|  | -(-k4\_N6\*P\_N6) |

# Model Equations

## ODEs

|  | ODEs |
| --- | --- |
| 1 | d(mRNA\_N1)/dt = (k0\_N1+k1\_N1\*[HILL\_N1|-N6]\*[HILL\_N1|-N4]) - (-(-k2\_N1\*mRNA\_N1)) |
| 2 | d(P\_N1)/dt = (k3\_N1\*mRNA\_N1) - (-(-k4\_N1\*P\_N1)) |
| 3 | d(mRNA\_N2)/dt = (k0\_N2+k1\_N2\*[HILL\_N2|-N1]\*[HILL\_N2|-N5]) - (-(-k2\_N2\*mRNA\_N2)) |
| 4 | d(P\_N2)/dt = (k3\_N2\*mRNA\_N2) - (-(-k4\_N2\*P\_N2)) |
| 5 | d(mRNA\_N3)/dt = (k0\_N3+k1\_N3\*[HILL\_N3|-N2]\*[HILL\_N3|-N6]) - (-(-k2\_N3\*mRNA\_N3)) |
| 6 | d(P\_N3)/dt = (k3\_N3\*mRNA\_N3) - (-(-k4\_N3\*P\_N3)) |
| 7 | d(mRNA\_N4)/dt = (k0\_N4+k1\_N4\*[HILL\_N4|-N3]\*[HILL\_N4|-N1]) - (-(-k2\_N4\*mRNA\_N4)) |
| 8 | d(P\_N4)/dt = (k3\_N4\*mRNA\_N4) - (-(-k4\_N4\*P\_N4)) |
| 9 | d(mRNA\_N5)/dt = (k0\_N5+k1\_N5\*[HILL\_N5|-N4]\*[HILL\_N5|-N2]) - (-(-k2\_N5\*mRNA\_N5)) |
| 10 | d(P\_N5)/dt = (k3\_N5\*mRNA\_N5) - (-(-k4\_N5\*P\_N5)) |
| 11 | d(mRNA\_N6)/dt = (k0\_N6+k1\_N6\*[HILL\_N6|-N5]\*[HILL\_N6|-N3]) - (-(-k2\_N6\*mRNA\_N6)) |
| 12 | d(P\_N6)/dt = (k3\_N6\*mRNA\_N6) - (-(-k4\_N6\*P\_N6)) |

Report generated by SimBiology v. 23.2 (R2023b) on 08-Aug-2024 15:17:05
