# Supplementary material for: A tool for modeling gene regulatory networks (GRN_modeler) and its applications to synthetic biology
Source: Mol Syst Biol. 2025 Sep 29;21(11):1618–37. doi: 10.1038/s44320-025-00148-8 (PMC12583811; doi:10.1038/s44320-025-00148-8)
Supplement: Supplementary file 2 — HTML model files [file 44320_2025_148_MOESM2_ESM.zip › SI/N6_A135.html]

GRN


# Model: GRN

## Quantities

|  | Quantity Name | Type | Scope | Value | Initial Value | Units | Notes |
| --- | --- | --- | --- | --- | --- | --- | --- |
| 1 | Ecoli | compartment | GRN | 0.7 | 0.7 | micrometer^3 |  |
| 2 | mRNA\_N1 | species | Ecoli | 0 | 0 | molecule | Individual |
| 3 | P\_N1 | species | Ecoli | 100 | 100 | molecule | Individual |
| 4 | mRNA\_N2 | species | Ecoli | 0 | 0 | molecule | Individual |
| 5 | P\_N2 | species | Ecoli | 0 | 0 | molecule | Individual |
| 6 | mRNA\_N3 | species | Ecoli | 0 | 0 | molecule | Individual |
| 7 | P\_N3 | species | Ecoli | 0 | 0 | molecule | Individual |
| 8 | mRNA\_N4 | species | Ecoli | 0 | 0 | molecule | Individual |
| 9 | P\_N4 | species | Ecoli | 0 | 0 | molecule | Individual |
| 10 | mRNA\_N5 | species | Ecoli | 0 | 0 | molecule | Individual |
| 11 | P\_N5 | species | Ecoli | 0 | 0 | molecule | Individual |
| 12 | mRNA\_N6 | species | Ecoli | 0 | 0 | molecule | Individual |
| 13 | P\_N6 | species | Ecoli | 0 | 0 | molecule | Individual |
| 14 | k0\_N1 | parameter | GRN | 0.03 | 0.03 | molecule/minute | Individual |
| 15 | k1\_N1 | parameter | GRN | 30 | 30 | molecule/minute | Individual |
| 16 | k2\_N1 | parameter | GRN | 0.34657 | 0.34657 | 1/minute | Individual |
| 17 | k3\_N1 | parameter | GRN | 6.9315 | 6.9315 | 1/minute | Individual |
| 18 | k4\_N1 | parameter | GRN | 0.069315 | 0.069315 | 1/minute | Individual |
| 19 | k0\_N2 | parameter | GRN | 0.03 | 0.03 | molecule/minute | Individual |
| 20 | k1\_N2 | parameter | GRN | 30 | 30 | molecule/minute | Individual |
| 21 | k2\_N2 | parameter | GRN | 0.34657 | 0.34657 | 1/minute | Individual |
| 22 | k3\_N2 | parameter | GRN | 6.9315 | 6.9315 | 1/minute | Individual |
| 23 | k4\_N2 | parameter | GRN | 0.069315 | 0.069315 | 1/minute | Individual |
| 24 | k0\_N3 | parameter | GRN | 0.03 | 0.03 | molecule/minute | Individual |
| 25 | k1\_N3 | parameter | GRN | 30 | 30 | molecule/minute | Individual |
| 26 | k2\_N3 | parameter | GRN | 0.34657 | 0.34657 | 1/minute | Individual |
| 27 | k3\_N3 | parameter | GRN | 6.9315 | 6.9315 | 1/minute | Individual |
| 28 | k4\_N3 | parameter | GRN | 0.069315 | 0.069315 | 1/minute | Individual |
| 29 | k0\_N4 | parameter | GRN | 0.03 | 0.03 | molecule/minute | Individual |
| 30 | k1\_N4 | parameter | GRN | 30 | 30 | molecule/minute | Individual |
| 31 | k2\_N4 | parameter | GRN | 0.34657 | 0.34657 | 1/minute | Individual |
| 32 | k3\_N4 | parameter | GRN | 6.9315 | 6.9315 | 1/minute | Individual |
| 33 | k4\_N4 | parameter | GRN | 0.069315 | 0.069315 | 1/minute | Individual |
| 34 | k0\_N5 | parameter | GRN | 0.03 | 0.03 | molecule/minute | Individual |
| 35 | k1\_N5 | parameter | GRN | 30 | 30 | molecule/minute | Individual |
| 36 | k2\_N5 | parameter | GRN | 0.34657 | 0.34657 | 1/minute | Individual |
| 37 | k3\_N5 | parameter | GRN | 6.9315 | 6.9315 | 1/minute | Individual |
| 38 | k4\_N5 | parameter | GRN | 0.069315 | 0.069315 | 1/minute | Individual |
| 39 | k0\_N6 | parameter | GRN | 0.03 | 0.03 | molecule/minute | Individual |
| 40 | k1\_N6 | parameter | GRN | 30 | 30 | molecule/minute | Individual |
| 41 | k2\_N6 | parameter | GRN | 0.34657 | 0.34657 | 1/minute | Individual |
| 42 | k3\_N6 | parameter | GRN | 6.9315 | 6.9315 | 1/minute | Individual |
| 43 | k4\_N6 | parameter | GRN | 0.069315 | 0.069315 | 1/minute | Individual |
| 44 | HILL\_N2<-N1 | parameter | GRN | 1 | 0.97504 | dimensionless | Individual |
| 45 | K\_N2<-N1 | parameter | GRN | 40 | 40 | molecule | Individual |
| 46 | n | parameter | GRN | 4 | 4 | dimensionless | Common |
| 47 | HILL\_N3|-N2 | parameter | GRN | 1 | 1 | dimensionless | Individual |
| 48 | K\_N3|-N2 | parameter | GRN | 40 | 40 | molecule | Individual |
| 49 | HILL\_N4<-N3 | parameter | GRN | 1 | 0 | dimensionless | Individual |
| 50 | K\_N4<-N3 | parameter | GRN | 40 | 40 | molecule | Individual |
| 51 | HILL\_N5|-N4 | parameter | GRN | 1 | 1 | dimensionless | Individual |
| 52 | K\_N5|-N4 | parameter | GRN | 40 | 40 | molecule | Individual |
| 53 | HILL\_N6<-N5 | parameter | GRN | 1 | 0 | dimensionless | Individual |
| 54 | K\_N6<-N5 | parameter | GRN | 40 | 40 | molecule | Individual |
| 55 | HILL\_N1|-N6 | parameter | GRN | 1 | 1 | dimensionless | Individual |
| 56 | K\_N1|-N6 | parameter | GRN | 40 | 40 | molecule | Individual |

## Repeated Assignments

|  | Repeated Assignments | Initial Value | Notes |
| --- | --- | --- | --- |
| 1 | [HILL\_N2<-N1] = (P\_N1/[K\_N2<-N1])^n/(1+(P\_N1/[K\_N2<-N1])^n) | 0.97504 | Individual |
| 2 | [HILL\_N3|-N2] = 1/(1+(P\_N2/[K\_N3|-N2])^n) | 1 | Individual |
| 3 | [HILL\_N4<-N3] = (P\_N3/[K\_N4<-N3])^n/(1+(P\_N3/[K\_N4<-N3])^n) | 0 | Individual |
| 4 | [HILL\_N5|-N4] = 1/(1+(P\_N4/[K\_N5|-N4])^n) | 1 | Individual |
| 5 | [HILL\_N6<-N5] = (P\_N5/[K\_N6<-N5])^n/(1+(P\_N5/[K\_N6<-N5])^n) | 0 | Individual |
| 6 | [HILL\_N1|-N6] = 1/(1+(P\_N6/[K\_N1|-N6])^n) | 1 | Individual |

## Reactions

|  | Reactions | Notes |
| --- | --- | --- |
| 1 | null <-> mRNA\_N1 | Individual |
|  | k0\_N1+k1\_N1\*[HILL\_N1|-N6]-k2\_N1\*mRNA\_N1 |  |
| 2 | null <-> P\_N1 | Individual |
|  | k3\_N1\*mRNA\_N1-k4\_N1\*P\_N1 |  |
| 3 | null <-> mRNA\_N2 | Individual |
|  | k0\_N2+k1\_N2\*[HILL\_N2<-N1]-k2\_N2\*mRNA\_N2 |  |
| 4 | null <-> P\_N2 | Individual |
|  | k3\_N2\*mRNA\_N2-k4\_N2\*P\_N2 |  |
| 5 | null <-> mRNA\_N3 | Individual |
|  | k0\_N3+k1\_N3\*[HILL\_N3|-N2]-k2\_N3\*mRNA\_N3 |  |
| 6 | null <-> P\_N3 | Individual |
|  | k3\_N3\*mRNA\_N3-k4\_N3\*P\_N3 |  |
| 7 | null <-> mRNA\_N4 | Individual |
|  | k0\_N4+k1\_N4\*[HILL\_N4<-N3]-k2\_N4\*mRNA\_N4 |  |
| 8 | null <-> P\_N4 | Individual |
|  | k3\_N4\*mRNA\_N4-k4\_N4\*P\_N4 |  |
| 9 | null <-> mRNA\_N5 | Individual |
|  | k0\_N5+k1\_N5\*[HILL\_N5|-N4]-k2\_N5\*mRNA\_N5 |  |
| 10 | null <-> P\_N5 | Individual |
|  | k3\_N5\*mRNA\_N5-k4\_N5\*P\_N5 |  |
| 11 | null <-> mRNA\_N6 | Individual |
|  | k0\_N6+k1\_N6\*[HILL\_N6<-N5]-k2\_N6\*mRNA\_N6 |  |
| 12 | null <-> P\_N6 | Individual |
|  | k3\_N6\*mRNA\_N6-k4\_N6\*P\_N6 |  |

# Model Equations

## ODEs

|  | ODEs |
| --- | --- |
| 1 | d(mRNA\_N1)/dt = (k0\_N1+k1\_N1\*[HILL\_N1|-N6]-k2\_N1\*mRNA\_N1) |
| 2 | d(P\_N1)/dt = (k3\_N1\*mRNA\_N1-k4\_N1\*P\_N1) |
| 3 | d(mRNA\_N2)/dt = (k0\_N2+k1\_N2\*[HILL\_N2<-N1]-k2\_N2\*mRNA\_N2) |
| 4 | d(P\_N2)/dt = (k3\_N2\*mRNA\_N2-k4\_N2\*P\_N2) |
| 5 | d(mRNA\_N3)/dt = (k0\_N3+k1\_N3\*[HILL\_N3|-N2]-k2\_N3\*mRNA\_N3) |
| 6 | d(P\_N3)/dt = (k3\_N3\*mRNA\_N3-k4\_N3\*P\_N3) |
| 7 | d(mRNA\_N4)/dt = (k0\_N4+k1\_N4\*[HILL\_N4<-N3]-k2\_N4\*mRNA\_N4) |
| 8 | d(P\_N4)/dt = (k3\_N4\*mRNA\_N4-k4\_N4\*P\_N4) |
| 9 | d(mRNA\_N5)/dt = (k0\_N5+k1\_N5\*[HILL\_N5|-N4]-k2\_N5\*mRNA\_N5) |
| 10 | d(P\_N5)/dt = (k3\_N5\*mRNA\_N5-k4\_N5\*P\_N5) |
| 11 | d(mRNA\_N6)/dt = (k0\_N6+k1\_N6\*[HILL\_N6<-N5]-k2\_N6\*mRNA\_N6) |
| 12 | d(P\_N6)/dt = (k3\_N6\*mRNA\_N6-k4\_N6\*P\_N6) |

Report generated by SimBiology v. 23.2 (R2023b) on 18-Sep-2024 13:18:09
