# Supplementary material for: A tool for modeling gene regulatory networks (GRN_modeler) and its applications to synthetic biology
Source: Mol Syst Biol. 2025 Sep 29;21(11):1618–37. doi: 10.1038/s44320-025-00148-8 (PMC12583811; doi:10.1038/s44320-025-00148-8)
Supplement: Supplementary file 2 — HTML model files [file 44320_2025_148_MOESM2_ESM.zip › SI/repressilator_light.html]

GRN


# Model: GRN

## Quantities

|  | Quantity Name | Type | Scope | Value | Initial Value | Units | Notes |
| --- | --- | --- | --- | --- | --- | --- | --- |
| 1 | Ecoli | compartment | GRN | 0.7 | 0.7 | micrometer^3 |  |
| 2 | mRNA\_N1 | species | Ecoli | 0 | 0 | molecule | Individual |
| 3 | P\_N1 | species | Ecoli | 100 | 100 | molecule | Individual |
| 4 | mRNA\_N2 | species | Ecoli | 0 | 0 | molecule | Individual |
| 5 | P\_N2 | species | Ecoli | 0 | 0 | molecule | Individual |
| 6 | mRNA\_N3 | species | Ecoli | 0 | 0 | molecule | Individual |
| 7 | P\_N3 | species | Ecoli | 0 | 0 | molecule | Individual |
| 8 | Light | species | Ecoli | 0 | 50 | micromolarity | Individual |
| 9 | C | species | Ecoli | 0 | 0 | molecule |  |
| 10 | k0\_N1 | parameter | GRN | 0.03 | 0.03 | molecule/minute | Individual |
| 11 | k1\_N1 | parameter | GRN | 30 | 30 | molecule/minute | Individual |
| 12 | k2\_N1 | parameter | GRN | 0.34657 | 0.34657 | 1/minute | Individual |
| 13 | k3\_N1 | parameter | GRN | 6.9315 | 6.9315 | 1/minute | Individual |
| 14 | k4\_N1 | parameter | GRN | 0.069315 | 0.069315 | 1/minute | Individual |
| 15 | k0\_N2 | parameter | GRN | 0.03 | 0.03 | molecule/minute | Individual |
| 16 | k1\_N2 | parameter | GRN | 30 | 30 | molecule/minute | Individual |
| 17 | k2\_N2 | parameter | GRN | 0.34657 | 0.34657 | 1/minute | Individual |
| 18 | k3\_N2 | parameter | GRN | 6.9315 | 6.9315 | 1/minute | Individual |
| 19 | k4\_N2 | parameter | GRN | 0.069315 | 0.069315 | 1/minute | Individual |
| 20 | k0\_N3 | parameter | GRN | 0.03 | 0.03 | molecule/minute | Individual |
| 21 | k1\_N3 | parameter | GRN | 30 | 30 | molecule/minute | Individual |
| 22 | k2\_N3 | parameter | GRN | 0.34657 | 0.34657 | 1/minute | Individual |
| 23 | k3\_N3 | parameter | GRN | 6.9315 | 6.9315 | 1/minute | Individual |
| 24 | k4\_N3 | parameter | GRN | 0.069315 | 0.069315 | 1/minute | Individual |
| 25 | HILL\_N2|-N1 | parameter | GRN | 1 | 0.13793 | dimensionless | Individual |
| 26 | K\_N2|-N1 | parameter | GRN | 40 | 40 | molecule | Individual |
| 27 | n | parameter | GRN | 2 | 2 | dimensionless | Common |
| 28 | HILL\_N3|-N2 | parameter | GRN | 1 | 1 | dimensionless | Individual |
| 29 | K\_N3|-N2 | parameter | GRN | 40 | 40 | molecule | Individual |
| 30 | HILL\_N1|-N3 | parameter | GRN | 1 | 1 | dimensionless | Individual |
| 31 | K\_N1|-N3 | parameter | GRN | 40 | 40 | molecule | Individual |
| 32 | HILL\_N1<-Light | parameter | GRN | 1 | 0.60976 | dimensionless | Individual |
| 33 | K\_out\_N1<-Light | parameter | GRN | 40 | 40 | micromolarity | Individual |
| 34 | n\_out\_N1<-Light | parameter | GRN | 2 | 2 | dimensionless | Individual |
| 35 | A | parameter | GRN | 100 | 100 |  | Individual |
| 36 | T | parameter | GRN | 256 | 256 |  | Individual |
| 37 | C\_unit | parameter | GRN | 1 | 1 | molecule |  |
| 38 | k\_cell | parameter | GRN | 0.1 | 0.1 | 1/(molecule\*minute) |  |

## Repeated Assignments

|  | Repeated Assignments | Initial Value | Notes |
| --- | --- | --- | --- |
| 1 | [HILL\_N2|-N1] = 1/(1+(P\_N1/[K\_N2|-N1])^n) | 0.13793 | Individual |
| 2 | [HILL\_N3|-N2] = 1/(1+(P\_N2/[K\_N3|-N2])^n) | 1 | Individual |
| 3 | [HILL\_N1|-N3] = 1/(1+(P\_N3/[K\_N1|-N3])^n) | 1 | Individual |
| 4 | [HILL\_N1<-Light] = (Light/[K\_out\_N1<-Light])^[n\_out\_N1<-Light]/(1+(Light/[K\_out\_N1<-Light])^[n\_out\_N1<-Light]) | 0.60976 | Individual |
| 5 | Light = A\*(1+sin(2\*pi/T\*time))/2 | 50 |  |

## Reactions

|  | Reactions | Notes |
| --- | --- | --- |
| 1 | null <-> mRNA\_N1 | Individual |
|  | k0\_N1+k1\_N1\*[HILL\_N1|-N3]\*[HILL\_N1<-Light]-k2\_N1\*mRNA\_N1 |  |
| 2 | null <-> P\_N1 | Individual |
|  | k3\_N1\*mRNA\_N1-k4\_N1\*P\_N1 |  |
| 3 | null <-> mRNA\_N2 | Individual |
|  | k0\_N2+k1\_N2\*[HILL\_N2|-N1]-k2\_N2\*mRNA\_N2 |  |
| 4 | null <-> P\_N2 | Individual |
|  | k3\_N2\*mRNA\_N2-k4\_N2\*P\_N2 |  |
| 5 | null <-> mRNA\_N3 | Individual |
|  | k0\_N3+k1\_N3\*[HILL\_N3|-N2]-k2\_N3\*mRNA\_N3 |  |
| 6 | null <-> P\_N3 | Individual |
|  | k3\_N3\*mRNA\_N3-k4\_N3\*P\_N3 |  |
| 7 | null -> C |  |
|  | k\_cell\*C\*(C\_unit-C) |  |

# Model Equations

## ODEs

|  | ODEs |
| --- | --- |
| 1 | d(mRNA\_N1)/dt = (k0\_N1+k1\_N1\*[HILL\_N1|-N3]\*[HILL\_N1<-Light]-k2\_N1\*mRNA\_N1) |
| 2 | d(P\_N1)/dt = (k3\_N1\*mRNA\_N1-k4\_N1\*P\_N1) |
| 3 | d(mRNA\_N2)/dt = (k0\_N2+k1\_N2\*[HILL\_N2|-N1]-k2\_N2\*mRNA\_N2) |
| 4 | d(P\_N2)/dt = (k3\_N2\*mRNA\_N2-k4\_N2\*P\_N2) |
| 5 | d(mRNA\_N3)/dt = (k0\_N3+k1\_N3\*[HILL\_N3|-N2]-k2\_N3\*mRNA\_N3) |
| 6 | d(P\_N3)/dt = (k3\_N3\*mRNA\_N3-k4\_N3\*P\_N3) |
| 7 | d(C)/dt = (k\_cell\*C\*(C\_unit-C)) |

Report generated by SimBiology v. 23.2 (R2023b) on 03-Apr-2025 14:33:14
