# Supplementary material for: A tool for modeling gene regulatory networks (GRN_modeler) and its applications to synthetic biology
Source: Mol Syst Biol. 2025 Sep 29;21(11):1618–37. doi: 10.1038/s44320-025-00148-8 (PMC12583811; doi:10.1038/s44320-025-00148-8)
Supplement: Supplementary file 2 — HTML model files [file 44320_2025_148_MOESM2_ESM.zip › SI/stricker.html]

GRN


# Model: GRN

## Quantities

|  | Quantity Name | Type | Scope | Value | Initial Value | Units | Notes |
| --- | --- | --- | --- | --- | --- | --- | --- |
| 1 | Ecoli | compartment | GRN | 0.7 | 0.7 | micrometer^3 |  |
| 2 | DNA\_N1 | species | Ecoli | 30 | 30 | molecule | Individual |
| 3 | mRNA\_N1 | species | Ecoli | 0 | 0 | molecule | Individual |
| 4 | P\_N1 | species | Ecoli | 0 | 0 | molecule | Individual |
| 5 | DNA\_N2 | species | Ecoli | 30 | 30 | molecule | Individual |
| 6 | mRNA\_N2 | species | Ecoli | 0 | 0 | molecule | Individual |
| 7 | P\_N2 | species | Ecoli | 0 | 0 | molecule | Individual |
| 8 | dilution | parameter | GRN | 0.018 | 0.018 | 1/minute | Common |
| 9 | k\_P | parameter | GRN | 6.9315 | 6.9315 | 1/minute | Common |
| 10 | d\_P | parameter | GRN | 0.051315 | 0.051315 | 1/minute | Common |
| 11 | a0 | parameter | GRN | 0.03 | 0.03 | molecule/minute | Common |
| 12 | a1\_N1 | parameter | GRN | 1 | 1 | 1/minute | Individual |
| 13 | d\_RNA | parameter | GRN | 0.32857 | 0.32857 | 1/minute | Common |
| 14 | leak | parameter | GRN | 0 | 0 | dimensionless | Common |
| 15 | a1\_N2 | parameter | GRN | 1 | 1 | 1/minute | Individual |
| 16 | HILL\_N2<-N1 | parameter | GRN | 1 | 0 | dimensionless | Individual |
| 17 | K\_molecule\_N2<-N1 | parameter | GRN | 40 | 40 | molecule | Individual |
| 18 | n\_molecule\_N2<-N1 | parameter | GRN | 6 | 6 | dimensionless | Individual |
| 19 | HILL\_N1<-N1 | parameter | GRN | 1 | 0 | dimensionless | Individual |
| 20 | K\_molecule\_N1<-N1 | parameter | GRN | 40 | 40 | molecule | Individual |
| 21 | n\_molecule\_N1<-N1 | parameter | GRN | 2 | 2 | dimensionless | Individual |
| 22 | HILL\_N2|-N2 | parameter | GRN | 1 | 1 | dimensionless | Individual |
| 23 | K\_molecule\_N2|-N2 | parameter | GRN | 40 | 40 | molecule | Individual |
| 24 | n\_molecule\_N2|-N2 | parameter | GRN | 2 | 2 | dimensionless | Individual |
| 25 | HILL\_N1|-N2 | parameter | GRN | 1 | 1 | dimensionless | Individual |
| 26 | K\_molecule\_N1|-N2 | parameter | GRN | 40 | 40 | molecule | Individual |
| 27 | n\_molecule\_N1|-N2 | parameter | GRN | 6 | 6 | dimensionless | Individual |

## Repeated Assignments

|  | Repeated Assignments | Initial Value | Notes |
| --- | --- | --- | --- |
| 1 | [HILL\_N2<-N1] = (P\_N1/[K\_molecule\_N2<-N1])^[n\_molecule\_N2<-N1]/(1+(P\_N1/[K\_molecule\_N2<-N1])^[n\_molecule\_N2<-N1]) | 0 | Individual |
| 2 | [HILL\_N1<-N1] = (P\_N1/[K\_molecule\_N1<-N1])^[n\_molecule\_N1<-N1]/(1+(P\_N1/[K\_molecule\_N1<-N1])^[n\_molecule\_N1<-N1]) | 0 | Individual |
| 3 | [HILL\_N2|-N2] = 1/(1+(P\_N2/[K\_molecule\_N2|-N2])^[n\_molecule\_N2|-N2]) | 1 | Individual |
| 4 | [HILL\_N1|-N2] = 1/(1+(P\_N2/[K\_molecule\_N1|-N2])^[n\_molecule\_N1|-N2]) | 1 | Individual |

## Reactions

|  | Reactions | Notes |
| --- | --- | --- |
| 1 | null <-> mRNA\_N1 | Individual |
|  | a0+a1\_N1\*(leak+(1-leak)\*[HILL\_N1<-N1]\*[HILL\_N1|-N2])\*DNA\_N1-(dilution+d\_RNA)\*mRNA\_N1 |  |
| 2 | null <-> P\_N1 | Individual |
|  | k\_P\*mRNA\_N1-(dilution+d\_P)\*P\_N1 |  |
| 3 | null <-> mRNA\_N2 | Individual |
|  | a0+a1\_N2\*(leak+(1-leak)\*[HILL\_N2<-N1]\*[HILL\_N2|-N2])\*DNA\_N2-(dilution+d\_RNA)\*mRNA\_N2 |  |
| 4 | null <-> P\_N2 | Individual |
|  | k\_P\*mRNA\_N2-(dilution+d\_P)\*P\_N2 |  |

# Model Equations

## ODEs

|  | ODEs |
| --- | --- |
| 1 | d(mRNA\_N1)/dt = (a0+a1\_N1\*(leak+(1-leak)\*[HILL\_N1<-N1]\*[HILL\_N1|-N2])\*DNA\_N1-(dilution+d\_RNA)\*mRNA\_N1) |
| 2 | d(P\_N1)/dt = (k\_P\*mRNA\_N1-(dilution+d\_P)\*P\_N1) |
| 3 | d(mRNA\_N2)/dt = (a0+a1\_N2\*(leak+(1-leak)\*[HILL\_N2<-N1]\*[HILL\_N2|-N2])\*DNA\_N2-(dilution+d\_RNA)\*mRNA\_N2) |
| 4 | d(P\_N2)/dt = (k\_P\*mRNA\_N2-(dilution+d\_P)\*P\_N2) |

Report generated by SimBiology v. 23.2 (R2023b) on 26-Mar-2025 14:28:50
