# Supplementary material for: A tool for modeling gene regulatory networks (GRN_modeler) and its applications to synthetic biology
Source: Mol Syst Biol. 2025 Sep 29;21(11):1618–37. doi: 10.1038/s44320-025-00148-8 (PMC12583811; doi:10.1038/s44320-025-00148-8)
Supplement: Supplementary file 2 — HTML model files [file 44320_2025_148_MOESM2_ESM.zip › SI/toematzu.html]

GRN


# Model: GRN

## Quantities

|  | Quantity Name | Type | Scope | Value | Initial Value | Units | Notes |
| --- | --- | --- | --- | --- | --- | --- | --- |
| 1 | Ecoli | compartment | GRN | 0.7 | 0.7 | micrometer^3 |  |
| 2 | mRNA\_R1 | species | Ecoli | 0 | 0 | molecule | Individual |
| 3 | uP\_R1 | species | Ecoli | 0 | 0 | molecule | Individual |
| 4 | P\_R1 | species | Ecoli | 0 | 0 | molecule | Individual |
| 5 | mRNA\_R2 | species | Ecoli | 0 | 0 | molecule | Individual |
| 6 | uP\_R2 | species | Ecoli | 0 | 0 | molecule | Individual |
| 7 | P\_R2 | species | Ecoli | 100 | 100 | molecule | Individual |
| 8 | mRNA\_R3 | species | Ecoli | 0 | 0 | molecule | Individual |
| 9 | uP\_R3 | species | Ecoli | 0 | 0 | molecule | Individual |
| 10 | P\_R3 | species | Ecoli | 0 | 0 | molecule | Individual |
| 11 | mRNA\_G | species | Ecoli | 0 | 0 | molecule | Individual |
| 12 | uP\_G | species | Ecoli | 0 | 0 | molecule | Individual |
| 13 | P\_G | species | Ecoli | 0 | 0 | molecule | Individual |
| 14 | C | species | Ecoli | 100 | 100 | molecule | Individual |
| 15 | L | species | Ecoli | 300 | 300 | molecule | Individual |
| 16 | Y | species | Ecoli | 100 | 100 | molecule | Individual |
| 17 | I2 | species | Ecoli | 100 | 100 | micromolarity | Individual |
| 18 | U | species | Ecoli | 100 | 100 | molecule | Individual |
| 19 | I1 | species | Ecoli | 100 | 100 | micromolarity | Individual |
| 20 | n\_copy\_R1 | parameter | GRN | 25 | 25 | molecule | Individual |
| 21 | a0\_R1 | parameter | GRN | 0.001 | 0.001 | 1/minute | Individual |
| 22 | a1\_R1 | parameter | GRN | 100 | 100 | 1/minute | Individual |
| 23 | k\_mRNA\_degr | parameter | GRN | 0.5 | 0.5 | 1/minute | Common |
| 24 | k\_translation\_R1 | parameter | GRN | 50 | 50 | 1/minute | Individual |
| 25 | k\_mat | parameter | GRN | 0.4 | 0.4 | 1/minute | Common |
| 26 | dilution | parameter | GRN | 0.01 | 0.01 | 1/minute | Common |
| 27 | n\_copy\_R2 | parameter | GRN | 50 | 50 | molecule | Individual |
| 28 | a0\_R2 | parameter | GRN | 0.001 | 0.001 | 1/minute | Individual |
| 29 | a1\_R2 | parameter | GRN | 10000 | 10000 | 1/minute | Individual |
| 30 | k\_translation\_R2 | parameter | GRN | 50 | 50 | 1/minute | Individual |
| 31 | n\_copy\_R3 | parameter | GRN | 25 | 25 | molecule | Individual |
| 32 | a0\_R3 | parameter | GRN | 0.001 | 0.001 | 1/minute | Individual |
| 33 | a1\_R3 | parameter | GRN | 100 | 100 | 1/minute | Individual |
| 34 | k\_translation\_R3 | parameter | GRN | 50 | 50 | 1/minute | Individual |
| 35 | n\_copy\_G | parameter | GRN | 50 | 50 | molecule | Individual |
| 36 | a0\_G | parameter | GRN | 0.001 | 0.001 | 1/minute | Individual |
| 37 | a1\_G | parameter | GRN | 1000 | 1000 | 1/minute | Individual |
| 38 | k\_translation\_G | parameter | GRN | 50 | 50 | 1/minute | Individual |
| 39 | K\_protease | parameter | GRN | 30 | 30 | molecule | Common |
| 40 | k\_protease\_max | parameter | GRN | 50 | 50 | 1/minute | Common |
| 41 | protease\_rate\_C | parameter | GRN | 0 | 38.4615 | 1/minute | Individual |
| 42 | Substrates\_C | parameter | GRN | 0 | 100 | molecule | Individual |
| 43 | protease\_rate\_L | parameter | GRN | 0 | 500 | 1/minute | Individual |
| 44 | Substrates\_L | parameter | GRN | 0 | 0 | molecule | Individual |
| 45 | HILL\_R1|-R3 | parameter | GRN | 1 | 1 | dimensionless | Individual |
| 46 | K\_molecule\_R1|-R3 | parameter | GRN | 5 | 5 | molecule | Individual |
| 47 | n\_molecule\_R1|-R3 | parameter | GRN | 2 | 2 | dimensionless | Individual |
| 48 | HILL\_R2|-R1 | parameter | GRN | 1 | 1 | dimensionless | Individual |
| 49 | K\_molecule\_R2|-R1 | parameter | GRN | 5 | 5 | molecule | Individual |
| 50 | n\_molecule\_R2|-R1 | parameter | GRN | 2 | 2 | dimensionless | Individual |
| 51 | HILL\_R3|-R2 | parameter | GRN | 1 | 0.0024938 | dimensionless | Individual |
| 52 | K\_molecule\_R3|-R2 | parameter | GRN | 5 | 5 | molecule | Individual |
| 53 | n\_molecule\_R3|-R2 | parameter | GRN | 2 | 2 | dimensionless | Individual |
| 54 | HILL\_G|-R3 | parameter | GRN | 1 | 1 | dimensionless | Individual |
| 55 | K\_molecule\_G|-R3 | parameter | GRN | 5 | 5 | molecule | Individual |
| 56 | n\_molecule\_G|-R3 | parameter | GRN | 2 | 2 | dimensionless | Individual |
| 57 | HILL\_R2<-Y | parameter | GRN | 1 | 0.7191 | dimensionless | Individual |
| 58 | K\_molecule\_R2<-Y | parameter | GRN | 50 | 50 | molecule | Individual |
| 59 | n\_molecule\_R2<-Y | parameter | GRN | 2 | 2 | dimensionless | Individual |
| 60 | HILL\_R2<-Y<-I2 | parameter | GRN | 1 | 0.8 | dimensionless | Individual |
| 61 | K\_micromolarity\_R2<-Y<-I2 | parameter | GRN | 50 | 50 | micromolarity | Individual |
| 62 | n\_micromolarity\_R2<-Y<-I2 | parameter | GRN | 2 | 2 | dimensionless | Individual |
| 63 | HILL\_G<-U | parameter | GRN | 1 | 0.7191 | dimensionless | Individual |
| 64 | K\_molecule\_G<-U | parameter | GRN | 50 | 50 | molecule | Individual |
| 65 | n\_molecule\_G<-U | parameter | GRN | 2 | 2 | dimensionless | Individual |
| 66 | HILL\_G<-U<-I1 | parameter | GRN | 1 | 0.8 | dimensionless | Individual |
| 67 | K\_micromolarity\_G<-U<-I1 | parameter | GRN | 50 | 50 | micromolarity | Individual |
| 68 | n\_micromolarity\_G<-U<-I1 | parameter | GRN | 2 | 2 | dimensionless | Individual |

## Repeated Assignments

|  | Repeated Assignments | Initial Value | Notes |
| --- | --- | --- | --- |
| 1 | protease\_rate\_C = k\_protease\_max\*C/(K\_protease+Substrates\_C) | 38.4615 | Individual |
| 2 | Substrates\_C = uP\_R1+P\_R1+uP\_R2+P\_R2+uP\_R3+P\_R3 | 100 | Individual |
| 3 | protease\_rate\_L = k\_protease\_max\*L/(K\_protease+Substrates\_L) | 500 | Individual |
| 4 | Substrates\_L = uP\_G+P\_G | 0 | Individual |
| 5 | [HILL\_R1|-R3] = 1/(1+(P\_R3/[K\_molecule\_R1|-R3])^[n\_molecule\_R1|-R3]) | 1 | Individual |
| 6 | [HILL\_R2|-R1] = 1/(1+(P\_R1/[K\_molecule\_R2|-R1])^[n\_molecule\_R2|-R1]) | 1 | Individual |
| 7 | [HILL\_R3|-R2] = 1/(1+(P\_R2/[K\_molecule\_R3|-R2])^[n\_molecule\_R3|-R2]) | 0.0024938 | Individual |
| 8 | [HILL\_G|-R3] = 1/(1+(P\_R3/[K\_molecule\_G|-R3])^[n\_molecule\_G|-R3]) | 1 | Individual |
| 9 | [HILL\_R2<-Y] = (Y\*[HILL\_R2<-Y<-I2]/[K\_molecule\_R2<-Y])^[n\_molecule\_R2<-Y]/(1+(Y\*[HILL\_R2<-Y<-I2]/[K\_molecule\_R2<-Y])^[n\_molecule\_R2<-Y]) | 0.7191 | Individual |
| 10 | [HILL\_R2<-Y<-I2] = (I2/[K\_micromolarity\_R2<-Y<-I2])^[n\_micromolarity\_R2<-Y<-I2]/(1+(I2/[K\_micromolarity\_R2<-Y<-I2])^[n\_micromolarity\_R2<-Y<-I2]) | 0.8 | Individual |
| 11 | [HILL\_G<-U] = (U\*[HILL\_G<-U<-I1]/[K\_molecule\_G<-U])^[n\_molecule\_G<-U]/(1+(U\*[HILL\_G<-U<-I1]/[K\_molecule\_G<-U])^[n\_molecule\_G<-U]) | 0.7191 | Individual |
| 12 | [HILL\_G<-U<-I1] = (I1/[K\_micromolarity\_G<-U<-I1])^[n\_micromolarity\_G<-U<-I1]/(1+(I1/[K\_micromolarity\_G<-U<-I1])^[n\_micromolarity\_G<-U<-I1]) | 0.8 | Individual |

## Reactions

|  | Reactions | Notes |
| --- | --- | --- |
| 1 | null <-> mRNA\_R1 | Individual |
|  | n\_copy\_R1\*(a0\_R1+a1\_R1\*[HILL\_R1|-R3])-(k\_mRNA\_degr+dilution)\*mRNA\_R1 |  |
| 2 | null <-> uP\_R1 | Individual |
|  | k\_translation\_R1\*mRNA\_R1-(dilution)\*uP\_R1 |  |
| 3 | uP\_R1 -> P\_R1 | Individual |
|  | k\_mat\*uP\_R1 |  |
| 4 | null <-> P\_R1 | Individual |
|  | -(dilution)\*P\_R1 |  |
| 5 | null <-> mRNA\_R2 | Individual |
|  | n\_copy\_R2\*(a0\_R2+a1\_R2\*[HILL\_R2|-R1]\*[HILL\_R2<-Y])-(k\_mRNA\_degr+dilution)\*mRNA\_R2 |  |
| 6 | null <-> uP\_R2 | Individual |
|  | k\_translation\_R2\*mRNA\_R2-(dilution)\*uP\_R2 |  |
| 7 | uP\_R2 -> P\_R2 | Individual |
|  | k\_mat\*uP\_R2 |  |
| 8 | null <-> P\_R2 | Individual |
|  | -(dilution)\*P\_R2 |  |
| 9 | null <-> mRNA\_R3 | Individual |
|  | n\_copy\_R3\*(a0\_R3+a1\_R3\*[HILL\_R3|-R2])-(k\_mRNA\_degr+dilution)\*mRNA\_R3 |  |
| 10 | null <-> uP\_R3 | Individual |
|  | k\_translation\_R3\*mRNA\_R3-(dilution)\*uP\_R3 |  |
| 11 | uP\_R3 -> P\_R3 | Individual |
|  | k\_mat\*uP\_R3 |  |
| 12 | null <-> P\_R3 | Individual |
|  | -(dilution)\*P\_R3 |  |
| 13 | null <-> mRNA\_G | Individual |
|  | n\_copy\_G\*(a0\_G+a1\_G\*[HILL\_G|-R3]\*[HILL\_G<-U])-(k\_mRNA\_degr+dilution)\*mRNA\_G |  |
| 14 | null <-> uP\_G | Individual |
|  | k\_translation\_G\*mRNA\_G-(dilution)\*uP\_G |  |
| 15 | uP\_G -> P\_G | Individual |
|  | k\_mat\*uP\_G |  |
| 16 | null <-> P\_G | Individual |
|  | -(dilution)\*P\_G |  |
| 17 | uP\_R1 -> null | Individual |
|  | protease\_rate\_C\*uP\_R1 |  |
| 18 | P\_R1 -> null | Individual |
|  | protease\_rate\_C\*P\_R1 |  |
| 19 | uP\_R2 -> null | Individual |
|  | protease\_rate\_C\*uP\_R2 |  |
| 20 | P\_R2 -> null | Individual |
|  | protease\_rate\_C\*P\_R2 |  |
| 21 | uP\_R3 -> null | Individual |
|  | protease\_rate\_C\*uP\_R3 |  |
| 22 | P\_R3 -> null | Individual |
|  | protease\_rate\_C\*P\_R3 |  |
| 23 | uP\_G -> null | Individual |
|  | protease\_rate\_L\*uP\_G |  |
| 24 | P\_G -> null | Individual |
|  | protease\_rate\_L\*P\_G |  |

# Model Equations

## ODEs

|  | ODEs |
| --- | --- |
| 1 | d(mRNA\_R1)/dt = (n\_copy\_R1\*(a0\_R1+a1\_R1\*[HILL\_R1|-R3])-(k\_mRNA\_degr+dilution)\*mRNA\_R1) |
| 2 | d(uP\_R1)/dt = (k\_translation\_R1\*mRNA\_R1-(dilution)\*uP\_R1) - (k\_mat\*uP\_R1) - (protease\_rate\_C\*uP\_R1) |
| 3 | d(P\_R1)/dt = (k\_mat\*uP\_R1) + (-(dilution)\*P\_R1) - (protease\_rate\_C\*P\_R1) |
| 4 | d(mRNA\_R2)/dt = (n\_copy\_R2\*(a0\_R2+a1\_R2\*[HILL\_R2|-R1]\*[HILL\_R2<-Y])-(k\_mRNA\_degr+dilution)\*mRNA\_R2) |
| 5 | d(uP\_R2)/dt = (k\_translation\_R2\*mRNA\_R2-(dilution)\*uP\_R2) - (k\_mat\*uP\_R2) - (protease\_rate\_C\*uP\_R2) |
| 6 | d(P\_R2)/dt = (k\_mat\*uP\_R2) + (-(dilution)\*P\_R2) - (protease\_rate\_C\*P\_R2) |
| 7 | d(mRNA\_R3)/dt = (n\_copy\_R3\*(a0\_R3+a1\_R3\*[HILL\_R3|-R2])-(k\_mRNA\_degr+dilution)\*mRNA\_R3) |
| 8 | d(uP\_R3)/dt = (k\_translation\_R3\*mRNA\_R3-(dilution)\*uP\_R3) - (k\_mat\*uP\_R3) - (protease\_rate\_C\*uP\_R3) |
| 9 | d(P\_R3)/dt = (k\_mat\*uP\_R3) + (-(dilution)\*P\_R3) - (protease\_rate\_C\*P\_R3) |
| 10 | d(mRNA\_G)/dt = (n\_copy\_G\*(a0\_G+a1\_G\*[HILL\_G|-R3]\*[HILL\_G<-U])-(k\_mRNA\_degr+dilution)\*mRNA\_G) |
| 11 | d(uP\_G)/dt = (k\_translation\_G\*mRNA\_G-(dilution)\*uP\_G) - (k\_mat\*uP\_G) - (protease\_rate\_L\*uP\_G) |
| 12 | d(P\_G)/dt = (k\_mat\*uP\_G) + (-(dilution)\*P\_G) - (protease\_rate\_L\*P\_G) |

Report generated by SimBiology v. 23.2 (R2023b) on 08-Aug-2024 14:36:46
