# Supplementary material for: A tool for modeling gene regulatory networks (GRN_modeler) and its applications to synthetic biology
Source: Mol Syst Biol. 2025 Sep 29;21(11):1618–37. doi: 10.1038/s44320-025-00148-8 (PMC12583811; doi:10.1038/s44320-025-00148-8)
Supplement: Supplementary file 12 — Source data Fig. 6 [file 44320_2025_148_MOESM12_ESM.zip › Figure 6/READme.rtf]

For both Figures 6e-f, only channel 2 (mCitrine fluorescence) was used.
