# Supplementary figures and images for: A tool for modeling gene regulatory networks (GRN_modeler) and its applications to synthetic biology
Source: Mol Syst Biol. 2025 Sep 29;21(11):1618–37. doi: 10.1038/s44320-025-00148-8 (PMC12583811; doi:10.1038/s44320-025-00148-8)

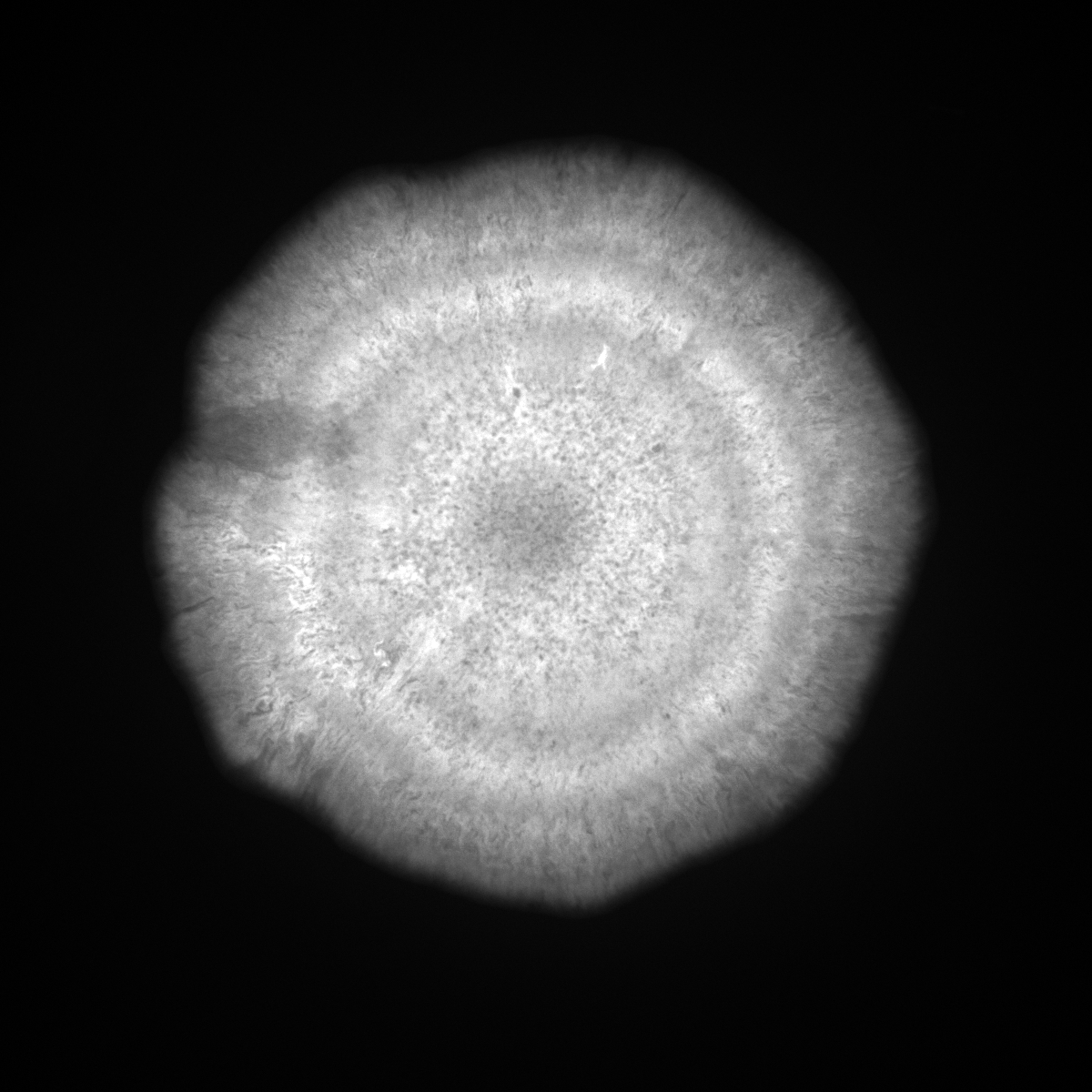

Supplement: Supplementary file 12 — Source data Fig. 6 [file 44320_2025_148_MOESM12_ESM.zip › Figure 6/6F_Sunlight activation_mCitrine.tif]

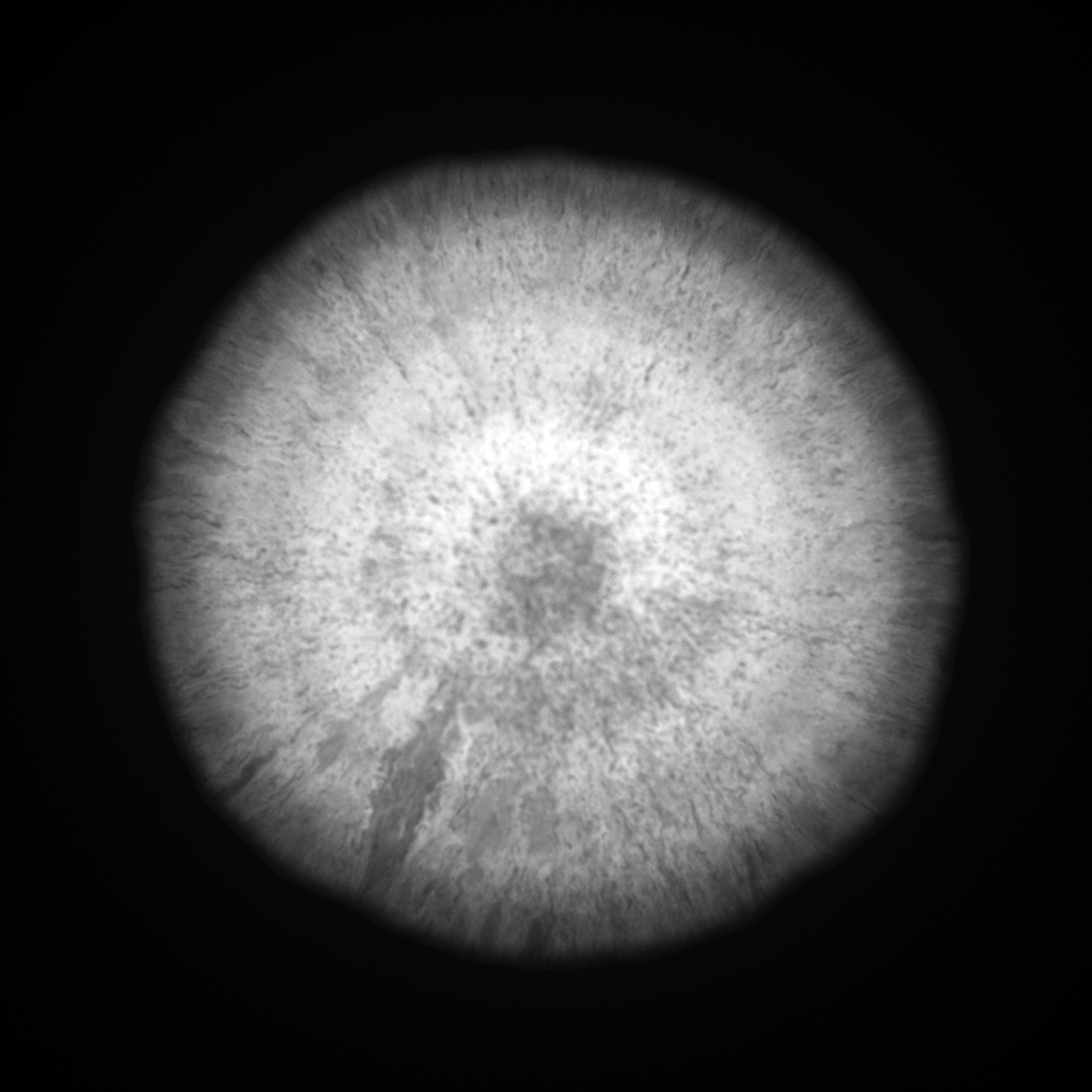

Supplement: Supplementary file 12 — Source data Fig. 6 [file 44320_2025_148_MOESM12_ESM.zip › Figure 6/6E_Light activation_mCitrine.tif]

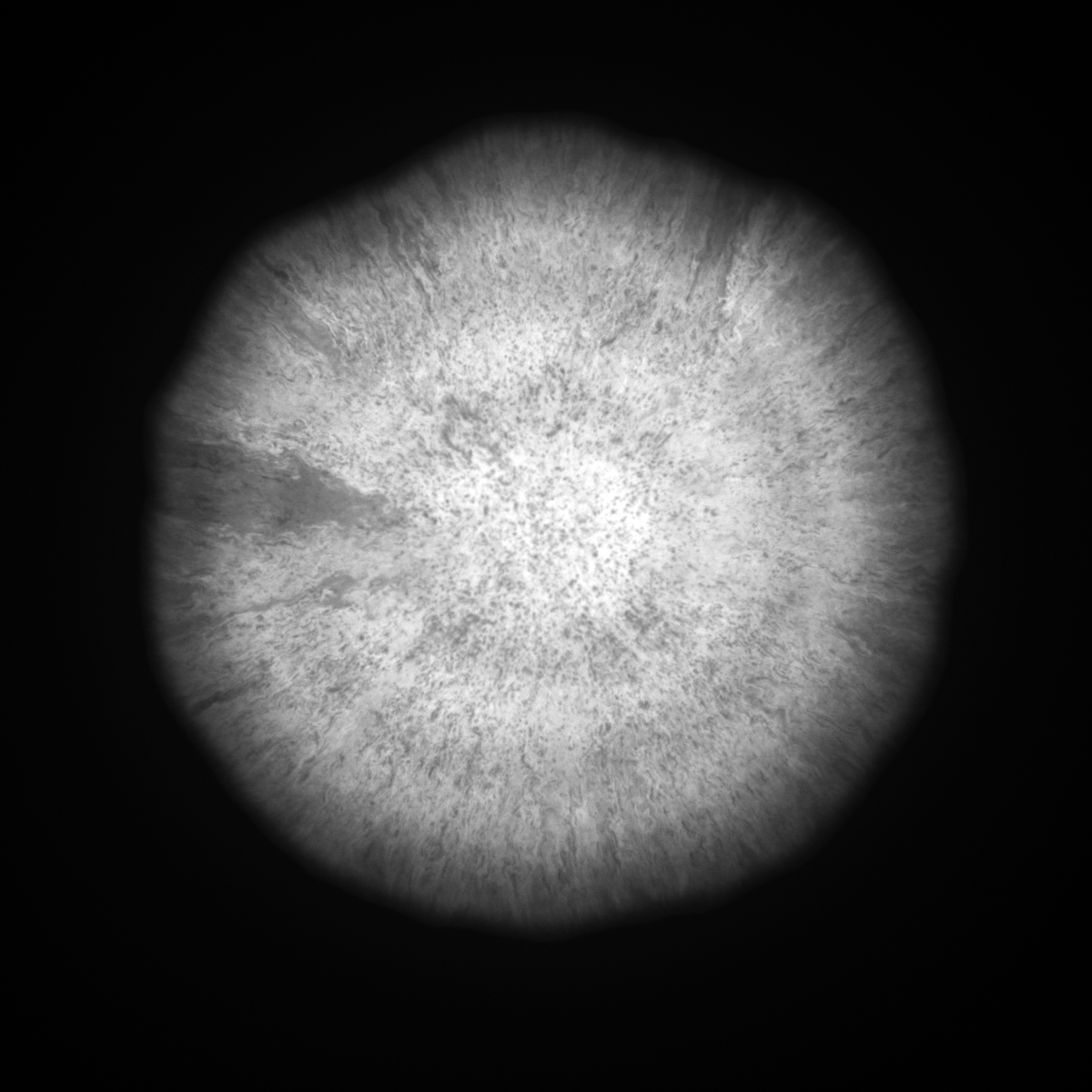

Supplement: Supplementary file 14 — Source data Fig. 8 [file 44320_2025_148_MOESM14_ESM.zip › Figure 8/8D_Light activation_mCitrine.tif]

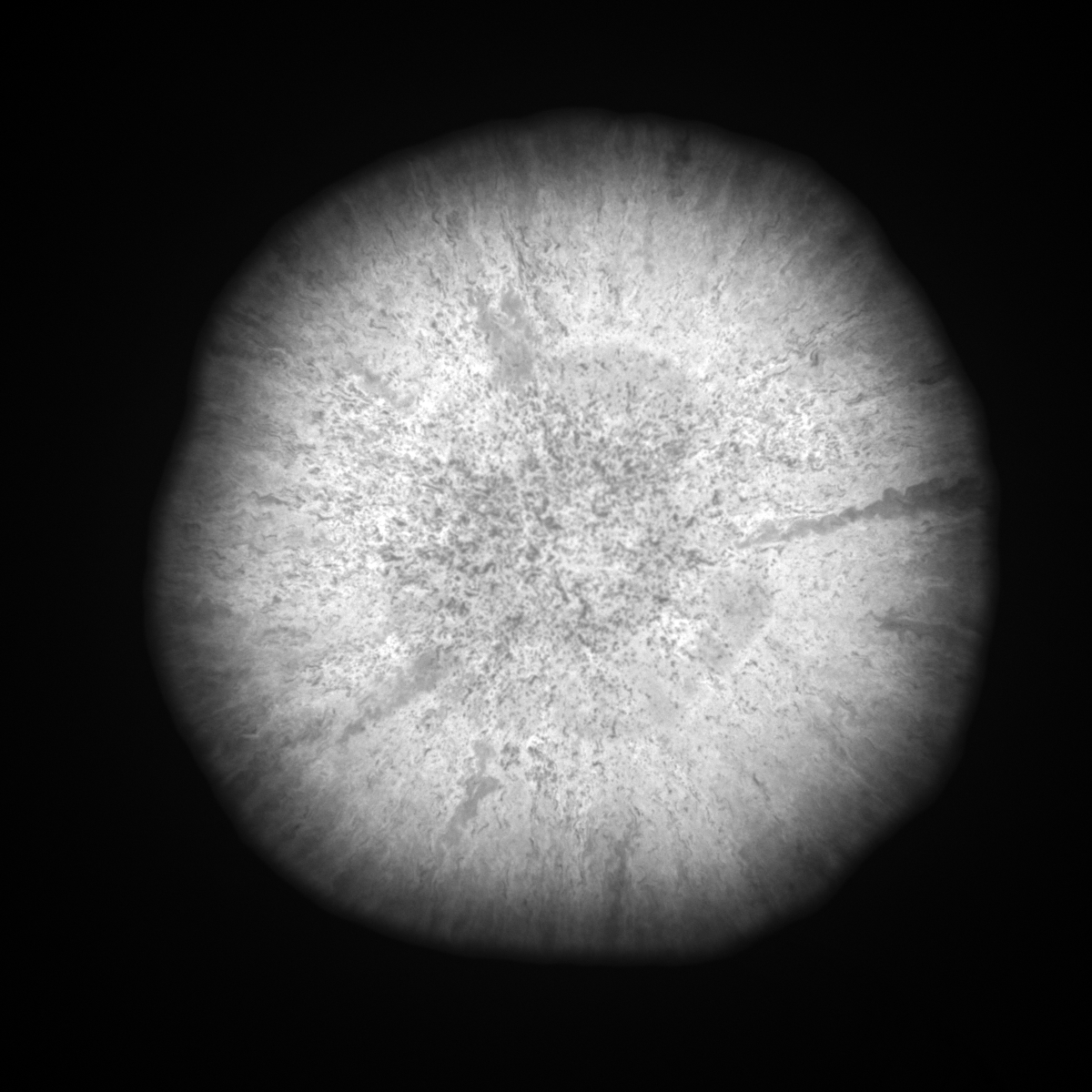

Supplement: Supplementary file 14 — Source data Fig. 8 [file 44320_2025_148_MOESM14_ESM.zip › Figure 8/8E_Light-Larab activation_mCitrine.tif]
