# Supplementary material for: A tool for modeling gene regulatory networks (GRN_modeler) and its applications to synthetic biology
Source: Mol Syst Biol. 2025 Sep 29;21(11):1618–37. doi: 10.1038/s44320-025-00148-8 (PMC12583811; doi:10.1038/s44320-025-00148-8)
Supplement: Supplementary file 14 — Source data Fig. 8 [file 44320_2025_148_MOESM14_ESM.zip › Figure 8/READme.rtf]

For both Figures 8d-e, only channel 2 (mCitrine fluorescence) was used.For the Figures 8d-e, we adjusted the minimum value of brightness (to 3400) to better visualize the rings. However, the data from the image analysis on Figure 8f was performed with the unmodified pictures.
